# Maternal smoking around birth and respiratory tract infection in adult offspring: A Mendelian randomization study

**DOI:** 10.18332/tid/226837

**Published:** 2026-10-02

**Authors:** Huan Ma, Yuanyuan Li, Hang Liang

**Affiliations:** 1 Institute of Medicine, Chongqing College of Humanities Science and Technology, Chongqing, China; 2 Department of Respiratory Medicine, Children’s Hospital of Chongqing Medical University, National Clinical Research Center for Child Health and Disorders, Ministry of Education Key Laboratory of Child Development and Disorders, Chongqing, China; 3 Chongqing Key Laboratory of Pediatric Metabolism and Inflammatory Diseases, Chongqing, China

**Keywords:** maternal smoking around birth, respiratory tract infection, pneumonia, adult offspring, Mendelian randomization

## Abstract

**INTRODUCTION:**

Maternal smoking during pregnancy impairs lung immune responses and leads to a higher frequency of respiratory infection in children. This study aims to assess the association of maternal smoking around birth with respiratory tract infection in adult offspring, using Mendelian randomization (MR).

**METHODS:**

This is an MR study based on the Integrative Epidemiology Unit (IEU) open Genome-Wide Association Study (GWAS) data. The exposure was maternal smoking around birth; the outcomes were upper respiratory tract infection (URTI), lower respiratory tract infection (LRTI), coronavirus disease 2019 (COVID-19), acute bronchitis, pneumonia, bacterial pneumonia, viral pneumonia, influenza pneumonia, and pneumonia death. The inverse variance weighted, weighted median, and MR-Egger methods were used as the main methods of MR analysis. Heterogeneity and pleiotropic analysis were performed to ensure the accuracy of the results.

**RESULTS:**

After removing single-nucleotide polymorphisms (SNPs) related to smoking by the offspring, maternal smoking around birth was associated with an increased risk of LRTI (OR=2.68; 95% CI: 1.29–5.58; p=0.008), COVID-19 (OR=2.32; 95% CI: 1.30–4.14; p=0.004), acute bronchitis (OR=7.14; 95% CI: 2.54–20.06; p<0.001), pneumonia (OR=1.90; 95% CI: 1.07–3.38; p=0.030), and bacterial pneumonia (OR=2.96; 95% CI: 1.14–7.69; p=0.026). No significant correlation was detected between maternal smoking and URTI (OR=4.29; 95% CI: 0.72–25.68; p=0.111).

**CONCLUSIONS:**

This Mendelian randomization study provides evidence that maternal smoking around birth is associated with an increased risk of LRTIs, particularly acute bronchitis, pneumonia, and bacterial pneumonia, along with COVID-19, in adult offspring. These findings suggest that the detrimental impact of early-life tobacco smoke exposure on respiratory health persists into adulthood, likely mediated by long-term alterations in pulmonary development and immune system programming.

## INTRODUCTION

Respiratory tract infection is defined as any infectious disease of the upper or lower respiratory tract^1^. It poses a major health problem and leads to high morbidity and short-term and long-term mortality worldwide^2,3^. According to the Global Burden of Diseases study in 2021, there were 12.8 billion incident cases of upper respiratory tract infection (URTI), and 344 million cases of lower respiratory tract infection (LRTI) globally, which contributed to a significant disease burden and was one of the leading causes of death worldwide^2,3^. Common pathogens of respiratory tract infection include *Respiratory syncytial virus* (RSV), *Rhinovirus*, *Hemophilus influenzae, Streptococcus pneumoniae, Staphylococcus aureus, Mycoplasma pneumoniae,* and severe acute respiratory syndrome (SARS) coronavirus 2 (SARS-CoV-2), which emerged in 2019^4^.

The central role of smoking in an individual’s respiratory diseases has been well established^5^. Despite various smoking cessation measures, smoking during pregnancy still remains prevalent worldwide; the prevalence was 8.1% in the European region, 5.9% in the region of the Americas, 1.2% in the Southeast Asian region, and 1.2% in the Western Pacific region^6^. Previous meta-analyses have demonstrated the association between smoke exposure during pregnancy and respiratory tract infection in the neonatal period^7^ and childhood^8^. A recent Mendelian randomization (MR) study demonstrated that maternal smoking around birth increased the risk of offspring developing chronic respiratory diseases, including asthma and chronic obstructive pulmonary disease (COPD), during adulthood, suggesting a continued impact of maternal smoking on lung health and development^9^. However, it remains uncertain whether maternal smoking around birth represents a strong determinant of respiratory tract infection until adulthood, because the available evidence is scarce.

MR analysis uses genetic variants as instrumental variables (IVs), which has advantages over other research methods^10^. Based on data from the largest available genome-wide association study (GWAS), this MR analysis was performed to explore the relationship between maternal smoking around birth and respiratory tract infection in offspring during adulthood.

## METHODS

This is an MR study based on publicly available GWAS data. The following basic assumptions constitute the premise of MR analysis. First, IVs must be intensely associated with the exposure factor. Second, IVs cannot be directly correlated to the outcome. Third, IVs must not be related to any potential confounding factors. The GWAS summary-level data used in this study were issued by the Integrative Epidemiology Unit (IEU) open GWAS project^11^. This study was exempt from the approval of the ethics committee because the data used in this study were public, anonymized, and de-identified.

### Study design

The exposure event was maternal smoking around birth. Outcome events were respiratory tract infection in adult offspring, including URTI, LRTI, and coronavirus disease 2019 (COVID-19). Given the positive association observed between maternal smoking and LRTI, further analyses were performed for LRTI subtypes, including acute bronchitis, pneumonia (due to any infectious pathogen), bacterial pneumonia, viral pneumonia, and influenza pneumonia. In addition, pneumonia death was included for assessing the disease severity.

### The selection of IVs

IVs were utilized as mediators to explore the causal relationship between exposure and outcome. IVs are generally genetic variations, among which single nucleotide polymorphisms (SNPs) are the most commonly used. SNPs associated with maternal smoking around birth were extracted from the IEU open GWAS project. IVs of p<5×10^-7^ were selected for MR analysis. The parameters used to eliminate linkage disequilibrium among variables were kb=10000 and r^2^=0.01. The F statistic was used to ensure a strong association between IVs and exposure; an F statistic >10 was generally considered to meet the requirements of strong association.

### Statistical analysis

The inverse variance weighted (IVW) method was conducted as the primary method for calculating the causal effect. In addition, we contrasted the consequences of the IVW method with the weighted median and the MR-Egger methods. The IVW model assumes the validity of all IVs and aggregates locus-specific Wald ratio estimates to quantify the effect of genetic traits on outcomes, which provides the most accurate results in the absence of heterogeneity or horizontal pleiotropy. In addition, forest plots and scatter plots were used to visualize the results.

Sensitivity analysis was performed using the following methods. First, Cochran’s Q test was used to detect heterogeneity. Second, the MR-Egger intercept test was used to determine horizontal pleiotropy. Third, the leave-one-out method was performed to assess whether there was a significant effect on the results after the removal of a single SNP. Fourth, the MR-Pleiotropy Residual Sum and Outlier method (MR-PRESSO) was used to detect outliers. A p<0.05 was used as the threshold for statistical significance. All the MR analyses were performed in R software (version 4.3.3)^12^ using the *TwoSampleMR* package.

## RESULTS

The associations between maternal smoking around birth and respiratory tract infections in offspring during adulthood were analyzed. GWAS summary-level data of maternal smoking around birth (397732 participants), URTI (486484 participants), LRTI (486484 participants), COVID-19 (1683768 participants), and pneumonia death (434005 participants) were extracted from the UK Biobank by the IEU open GWAS project. Data on acute bronchitis (216027 participants), pneumonia (218792 participants), bacterial pneumonia (196855 participants), viral pneumonia (189568 participants), and influenza pneumonia (218792 participants) were extracted from the FinnGen biobank by the IEU open GWAS project. More information about the exposure and outcome datasets is presented in Table 1.

**Table 1:** Characteristics of the exposure and outcome GWAS datasets used in the MR analysis

*IEU GWAS id*	*Publication year*	*Exposure or outcome*	*Participants European-descent individuals*	*Cases*	*Controls*
ukb-b-17685	2018	Maternal smoking around birth	397732	-	-
ieu-b-5063	2021	URTI	486484	2795	483689
ieu-b-4973	2021	LRTI	486484	14135	472349
ebi-a-GCST011073	2020	COVID-19	1683768	38984	1644784
finn-b-J10_BRONCHITIS	2021	Acute bronchitis	216027	7338	208689
finn-b-J10_PNEUMONIA	2021	Pneumonia	218792	27370	191422
finn-b-J10_PNEUMOBACT	2021	Bacterial pneumonia	196855	7987	188868
finn-b-J10_VIRALPNEUMO	2021	Viral pneumonia	189568	700	188868
finn-b-J10_INFLUPNEU	2021	Influenza pneumonia	218792	29924	188868
ieu-b-4979	2021	Pneumonia death	434005	3185	430820

This table presents the publicly available GWAS summary statistics utilized in this two-sample MR study. All datasets were obtained from the IEU OpenGWAS database including the UK Biobank and FinnGen study. For the exposure and each outcome, the GWAS ID, publication year, total sample size, and the number of cases and controls (where applicable) are provided. COVID-19: Coronavirus Disease 2019. GWAS: Genome-Wide Association Studies. IEU: Integrative Epidemiology Unit. MR: Mendelian randomization. URTI: upper respiratory tract infection. LRTI: lower respiratory tract infection.

### Selection of IVs

Following the selection process, the study obtained 1290 SNPs strongly correlated with the exposure variable. After pruning for linkage disequilibrium, a total of 45 SNPs for URTI, LRTI, and pneumonia death, and 44 SNPs for COVID-19, acute bronchitis, pneumonia, bacterial pneumonia, viral pneumonia, and influenza pneumonia, were included as IVs. All included SNPs had F-statistics >10, indicating the effectiveness of the selected IVs. Comprehensive details of IVs for each outcome are provided in Supplementary file Tables S1–S9.

### MR analysis before removing SNPs related to smoking by offspring

A total of 7 causalities were identified (p<0.05 by IVW method). Maternal smoking around birth was related to an increased risk of LRTI (OR=2.85; 95% CI: 1.50–5.38; p=0.001), COVID-19 (OR=1.98; 95% CI: 1.19–3.29; p=0.009), acute bronchitis (OR=5.77; 95% CI: 2.25–14.79; p<0.001), pneumonia (OR=2.29; 95%CI: 1.33–3.92; p=0.003), bacterial pneumonia (OR=3.62; 95% CI: 1.54–8.49; p=0.003), influenza pneumonia (OR=2.03; 95% CI: 1.19–3.47; p=0.009) and pneumonia death (OR=21.92; 95% CI: 5.90–81.48; p<0.001). The weighted median model further verified 2 of 7 associations: COVID-19 (OR=2.23; 95% CI: 1.07–4.62; p=0.032) and pneumonia death (OR=20.52; 95% CI: 2.99–140.63; p=0.002). None of the above associations was verified by the MR-Egger model (p>0.05). Moreover, all three analysis methods did not indicate any association of maternal smoking with URTI (IVW method: OR=4.06; 95% CI: 0.90–18.37; p=0.069) or viral pneumonia (IVW method: OR=2.32; 95% CI: 0.15–36.56; p=0.549). More results of the MR analysis are given in Table 2. The results of the 7 positive causalities are visualized in Supplementary file Figures S1 and S2. Leave-one-out analysis indicated that all positive causalities were very robust (Supplementary file Figure S3).

**Table 2:** The results of MR analysis before removing SNPs related to smoking by offspring

*Outcome*	*nSNPs*	*IVW method*		*Weighted median method*		*MR-Egger meth*	*od*	*Cochrane’s Q test*		*Pleiotropy (MR-Egger)*		
		*OR (95% CI)*	*p*	*OR (95%CI)*	*p*	*OR (95% CI)*	*p*	*Q*	*p*	*Intercept*	*SE*	*p*
URTI	45	4.06 (0.90–18.37)	0.069	3.03 (0.36–25.27)	0.305	6.51 (0.01–3094.90)	0.555	50.78	0.224	-0.003	0.022	0.877
LRTI	45	2.85 (1.50–5.38)	0.001	2.12 (0.87–5.16)	0.097	0.38 (0.03–4.84)	0.463	45.38	0.414	0.015	0.009	0.117
COVID-19	44	1.98 (1.19–3.29)	0.009	2.23 (1.07–4.62)	0.032	1.48 (0.19–11.70)	0.711	45.73	0.360	0.002	0.007	0.779
Acute bronchitis	44	5.77 (2.25–14.79)	<0.001	3.33 (0.87–12.77)	0.080	3.73 (0.07–200.49)	0.520	50.68	0.196	0.003	0.014	0.827
Pneumonia	44	2.29 (1.33–3.92)	0.003	1.33 (0.63–2.80)	0.448	5.08 (0.53–49.05)	0.167	52.18	0.159	–0.006	0.008	0.480
Bacterial pneumonia	44	3.62 (1.54–8.49)	0.003	2.49 (0.71–8.70)	0.154	6.48 (0.18–234.38)	0.313	42.31	0.501	-0.004	0.013	0.745
Viral pneumonia	44	2.32 (0.15–36.56)	0.549	0.62 (0.01–30.41)	0.808	0.07 (7.06E-07–7616.08)	0.660	35.19	0.796	0.025	0.042	0.549
Influenza pneumonia	44	2.03 (1.19–3.47)	0.009	1.48 (0.72–3.06)	0.288	2.50 (0.26–23.88)	0.431	55.47	0.096	-0.002	0.008	0.854
Pneumonia death	45	21.92 (5.90–81.48)	<0.001	20.52 (2.99–140.63)	0.002	1.57 (0.01–312.87)	0.868	45.09	0.426	0.019	0.019	0.319

The relationships between maternal smoking around birth and respiratory outcomes in the adult offspring were assessed using a two-sample MR method. Inverse variance weighted (IVW) was used as the primary analysis, weighted median and MR-Egger methods were included as complementary analyses. Cochran’s Q test was used to detect heterogeneity, and the MR-Egger intercept test was applied to determine horizontal pleiotropy. A p<0.05 was used as the threshold for statistical significance. COVID-19: Coronavirus Disease 2019. MR: Mendelian randomization. nSNPs: number of Single-Nucleotide Polymorphisms. OR: odds ratio. CI: confidence interval. SE: standard error. URTI: upper respiratory tract infection. LRTI: lower respiratory tract infection.

In addition, the Cochran’s Q test indicated the absence of statistically significant heterogeneity, and the consequences of the MR-Egger intercept suggested no directional pleiotropy in all MR analyses (Table 2). There were no outliers in all MR-PRESSO results.

### MR analysis after removing SNPs related to smoking by offspring

Considering the offspring’s smoking status may affect their risk of developing respiratory tract infection and confound the observed causal relationships, SNPs related to smoking by offspring were regarded as unreliable IVs. Specifically, the genes rs6011779 and rs10226228 were associated with lifetime smoking index, rs36072649, rs56322375 and rs17133347 were associated with smoking initiation, rs12089815 related to smoking status, and rs75596189 related to cigarettes smoked per day; these 7 SNPs were removed, and the MR analyses were performed again. Therefore, a total of 38 SNPs for URTI, LRTI and pneumonia death, 37 SNPs for COVID-19, acute bronchitis, pneumonia, bacterial pneumonia, viral pneumonia and influenza pneumonia, were included as IVs.

A total of 6 causalities were identified (p<0.05 by IVW method). Maternal smoking around birth was related to an increased risk of LRTI (OR=2.68; 95% CI: 1.29–5.58; p=0.008), COVID-19 (OR=2.32; 95% CI: 1.30–4.14; p=0.004), acute bronchitis (OR=7.14; 95% CI: 2.54–20.06; p<0.001), pneumonia (OR=1.90; 95% CI: 1.07–3.38; p=0.030), bacterial pneumonia (OR=2.96; 95% CI: 1.14–7.69; p=0.026) and pneumonia death (OR=13.35; 95% CI: 3.11–57.41; p<0.001). 2 of the 6 causalities were further verified by the weighted median model (COVID-19, OR=2.37; 95% CI: 1.07–5.25; p=0.033; acute bronchitis, OR=4.75; 95% CI: 1.12–20.23; p=0.035). None of the above associations was verified by the MR-Egger model (p>0.05). All three analysis methods did not find any association of maternal smoking with URTI (IVW method, OR=4.29; 95% CI: 0.72–25.68; p=0.111), viral pneumonia (IVW method, OR=2.95; 95% CI: 0.13–64.97; p=0.492), or influenza pneumonia (IVW method, OR=1.71; 95% CI: 0.96–3.06; p=0.071). More results of MR analysis are given in Table 3. The results of the 6 positive causalities are visualized in Supplementary file Figures S4 and S5. Leave-one-out analysis indicated that all positive causalities were very robust (Supplementary file Figure S6).

**Table 3:** The results of MR analysis after removing SNP related to smoking by offspring

*Outcome*	*nSNPs*	*IVW method*		*Weighted median method*		*MR-Egger method*		*Cochrane’s Q test*		*Pleiotropy (MR-Egger)*		
		*OR (95% CI)*	*p*	*OR (95% CI)*	*p*	*OR (95% CI)*	*p*	*Q*	*p*	*Intercept*	*SE*	*p*
URTI	38	4.29 (0.72–25.68)	0.111	1.58 (0.14–18.55)	0.716	2.31 (1.00E-3–4179.28)	0.828	47.86	0.109	0.004	0.026	0.869
LRTI	38	2.68 (1.29–5.58)	0.008	1.60 (0.60–4.25)	0.344	0.22 (0.01–4.27)	0.325	40.21	0.330	0.018	0.010	0.097
COVID-19	37	2.32 (1.30–4.14)	0.004	2.37 (1.07–5.25)	0.033	4.75 (0.44–51.48)	0.208	38.89	0.341	-0.005	0.008	0.547
Acute bronchitis	37	7.14 (2.54–20.06)	<0.001	4.75 (1.12–20.23)	0.035	1.91 (0.02–151.19)	0.774	40.58	0.276	0.009	0.015	0.546
Pneumonia	37	1.90 (1.07–3.38)	0.030	1.28 (0.58–2.82)	0.547	2.80 (0.24–32.55)	0.417	39.92	0.300	-0.003	0.009	0.752
Bacterial pneumonia	37	2.96 (1.14–7.69)	0.026	1.72 (0.44–6.82)	0.439	9.02 (0.16–506.56)	0.292	34.22	0.554	-0.008	0.014	0.580
Viral pneumonia	37	2.95 (0.13–64.97)	0.492	0.78 (0.01–65.29)	0.910	0.01 (2.76E-08–5409.64)	0.511	31.68	0.674	0.039	0.046	0.400
Influenza pneumonia	37	1.71 (0.96–3.06)	0.071	1.14 (0.53–2.45)	0.738	1.46 (0.12–17.45)	0.766	44.02	0.168	0.001	0.009	0.900
Pneumonia death	38	13.35 (3.11–57.41)	<0.001	7.45 (0.98–56.74)	0.053	1.15 (3.00E-3–490.66)	0.964	37.35	0.453	0.017	0.021	0.419

The relationships between maternal smoking around birth and respiratory outcomes in the adult offspring were assessed using a two-sample MR method. Inverse variance weighted (IVW) was used as the primary analysis, weighted median and MR-Egger methods were included as complementary analyses. Cochran’s Q test was used to detect heterogeneity, and the MR-Egger intercept test was applied to determine horizontal pleiotropy. A p<0.05 was used as the threshold for statistical significance. COVID-19: Coronavirus Disease 2019. MR: Mendelian randomization. nSNPs: number of Single-Nucleotide Polymorphisms. OR: odds ratio. CI: confidence interval. SE: standard error. URTI: upper respiratory tract infection. LRTI: lower respiratory tract infection.

In addition, Cochran’s Q test indicated the absence of statistically significant heterogeneity, and the consequences of the MR-Egger intercept suggested no directional pleiotropy in all MR analyses (Table 3). There were no outliers in all MR-PRESSO results.

## DISCUSSION

To the best of our knowledge, this is the first MR study on the causal relationships between maternal smoking around birth and respiratory tract infection in the adult offspring. After eliminating the possible effects of individuals’ smoking status through the removal of related SNPs, results have indicated that: 1) maternal smoking around birth was related to an increased risk of LRTI (especially acute bronchitis, pneumonia, and bacterial pneumonia) and COVID-19 in offspring during adulthood; and 2) maternal smoking around birth was not associated with URTI, viral pneumonia, or influenza pneumonia.

Evidence regarding the detrimental effects of smoke exposure on lung health has been well established. There are a multitude of effects of tobacco smoke on the lung immune system, predisposing smoking individuals to respiratory infections. Briefly, smoking affects the functions of airway epithelium (IFN production, permeability and mucociliary clearance), local innate immune cell functions (alveolar macrophages and neutrophils) as well as adaptive immune mechanisms^13,14^.

Use of cigarettes during pregnancy not only affects the smoking mothers themselves, but it has also been shown to adversely affect fetal lung development and lead to a higher frequency and severity of respiratory tract infections in early life^7,8^. Specifically, *in utero* smoke-exposed children had an increased risk of hospitalization for RSV infection^15,16^, a higher prevalence of bronchitis^17^ and pneumonia^18,19^. However, evidence regarding the prevalence of respiratory tract infection until adulthood is limited. Previous studies demonstrated impaired adult lung function, namely FEV1, FVC, FEV1/EVC and FEF25–75, due to prenatal smoke exposure^20,21^. In this MR study, maternal smoking around birth has been shown to pose a possible causal effect on LRTI (especially acute bronchitis, pneumonia, and bacterial pneumonia) and COVID-19 in the adult offspring, suggesting a long-term impact of maternal smoking during pregnancy on the respiratory system defending against pathogens.

Supporting this, previous animal studies have demonstrated a long-lasting alteration in macrophage profile among the *in utero* tobacco-exposed offspring. The nicotine exposure in pregnant mice had induced a Th2polarized milieu in the neonatal lung at baseline and skewed the neonatal alveolar macrophage towards M2 activation, therefore impairing the phagocytic function^22^. Similarly, another study found that perinatal nicotine-exposed mice presented a long-lasting decrease of alveolar macrophages and interstitial monocytes/macrophages until adulthood^23^. Additionally, maternal smoking also impaired interferon production in offspring. Previous human cohort studies indicated a decreased level of cord blood interferon-g (IFNG) by prenatal smoke exposure, and suppressed IFNG levels through 11 years of age by continued environmental tobacco smoke exposure^24,25^. Similarly, a primate study identified a decline in the production of IFNG and IFNG-inducible T-cell chemoattractant by *in utero* smoke exposure^26^. IFNG has been well-known as a broad-spectrum anti-microbial agent. An important function of IFNG is to arm myeloid cells with microbiocidal properties^27^. By contrast, the specific antiviral mechanisms of IFNG are poorly understood. Of note, a previous mouse study has reported the antiviral properties of IFNG by demonstrating that the recombinant cytokine itself provides strong protection against SARS-CoV-2 challenge when administered intranasally^28^. Therefore, the existing evidence, combined with our findings, may support clinicians to improve their health education for women with pregnancy, and encourage their smoking cessation.

The mechanisms underpinning the maternal smoking-induced abnormal lung immunity are largely unknown, but epigenetic interactions with tobacco smoke most likely play a role. Previous cord blood studies have detected multiple genes differentially methylated in newborns in relation to maternal smoking during pregnancy^29-31^. Specifically, Hinz et al.^29^ found that maternal smoking/exposure to tobacco smoke during pregnancy was associated with reduced numbers of regulatory T cells (Treg) in cord blood, which was accompanied by TSDR (Treg-specific demethylated region) demethylation in the *FOXP3* gene. Other studies have detected the gene *GFI1*, which was previously reported as a main driver for many of the T-cell, eosinophil, and neutrophil-related pathway scores, was differentially methylated with regard to maternal smoking during pregnancy^30,31^. Nevertheless, to understand whether and how the impaired lung immune response persists later in adulthood, more longitudinal human studies and mechanistic research are needed.

Another finding of this MR study is that maternal smoking around birth was not associated with URTI. Previous evidence regarding the relationship of maternal smoking and URTI in childhood was controversial. Hashimoto et al.^32^ found a positive association between active maternal smoking during pregnancy and URTI in infants up to 1 year of age. However, after adjusting for potential confounders and maternal smoking in the postnatal period, this causal effect no longer existed^33^. A similar result has been reported in children up to the age of 8–12 years, with an estimated risk of 0.77 detected for the association of prenatal maternal smoking and URTI^34^. Nevertheless, URTI incidence in adult offspring is barely studied.

Moreover, this MR analysis indicated no significant association between maternal smoking around birth and influenza pneumonia or viral pneumonia in adult offspring. Previous human studies have been limited to the pediatric population. Several cohort studies have reported an increased risk of hospitalization for RSV infection in maternal smoke-exposed children^15,16^. In addition, a retrospective case-control study reported an association between maternal smoking and hospitalization due to influenza pneumonia in infants^35^, whereas another study found no association between maternal smoking and influenza infection in children aged 0–4 years^36^. Relevant evidence is also available from experimental animal studies. By inoculating influenza intranasally *in utero* nicotine-exposed mice, Cool et al.^23^ have observed increased disease severity in their early adulthood, characterized by greater weight loss, elevated neutrophil counts, and reduced alveolar macrophages. Given the current evidence, it remains unclear whether the association of maternal smoking with viral/influenza pneumonia persists into adulthood or diminishes with aging. Further large, population-based, long-term prospective cohort studies are warranted to address this relationship.

### Strengths and limitations

This study has some strengths and limitations. SNPs are used as IVs to infer the causality of maternal smoking around birth and respiratory tract infection; SNPs related to offspring smoking are excluded in this MR study, therefore effectively overcoming the bias caused by reverse causality and confounding. Sensitivity and pleiotropic analyses were performed to ensure the accuracy of MR analysis. European populations were used for both exposure and outcomes to avoid unnecessary bias.

However, the results of this study should be interpreted with caution due to several limitations. First, although a positive association between maternal smoking around birth and pneumonia death was detected, it yielded an implausibly large effect estimate (OR=13.35; 95% CI: 3.11–57.41). This exaggerated effect is plausibly attributable to the limited number of cases (n=3185) and the fact that pneumonia death is a rare binary outcome. Therefore, this specific finding is unlikely to represent a genuine causal relationship and is most probably a statistical artifact. Second, postnatal environmental factors (such as allergen and secondhand smoke exposure) that may confound the observed associations were not investigated, and the potential dynastic effects could not be fully eliminated in this MR analysis. Third, the exact timing of maternal smoking around birth was not clearly defined in this study. Therefore, it remains unknown which specific time window, such as a period during late pregnancy or around the time of delivery, contributed to the observed associations. Lastly, SNP selection in GWAS data may have increased sample overlap rates between exposure and outcomes. Nevertheless, given that the F-values were >10, the impact of sample overlap is considered minimal.

## CONCLUSIONS

This Mendelian randomization study provides evidence that maternal smoking around birth is associated with an increased risk of LRTIs, particularly acute bronchitis, pneumonia, and bacterial pneumonia, along with COVID-19, in adult offspring. These findings suggest that the detrimental impact of early-life tobacco smoke exposure on respiratory health persists into adulthood, likely mediated by long-term alterations in pulmonary development and immune system programming.

## Supplementary Material



## Data Availability

The datasets analyzed for this study can be found in the IEU open GWAS project (https://gwas.mrcieu.ac.uk/).
