# Supplementary material for: Maternal smoking around birth and respiratory tract infection in adult offspring: A Mendelian randomization study
Source: Tob Induc Dis. 2026 Oct 2;24:10.18332/tid/226837. doi: 10.18332/tid/226837 (PMC13640021; doi:10.18332/tid/226837)
Supplement: Supplementary file 1 [file TID-24-183-s1.pdf]

TID
Tobacco Induced Diseases

Supplementary file

© 2026 Ma H. et al.

DOI:
10.18332/tid/226837

The content has been provided by the author(s) and has not been reviewed, verified, or endorsed by European Publishing. It may not have undergone peer review. The views, opinions, and recommendations expressed are solely those of the author(s) and do not necessarily reflect the position of European Publishing. European Publishing accepts no responsibility or liability for any consequences arising from the use of, or reliance on, this content.

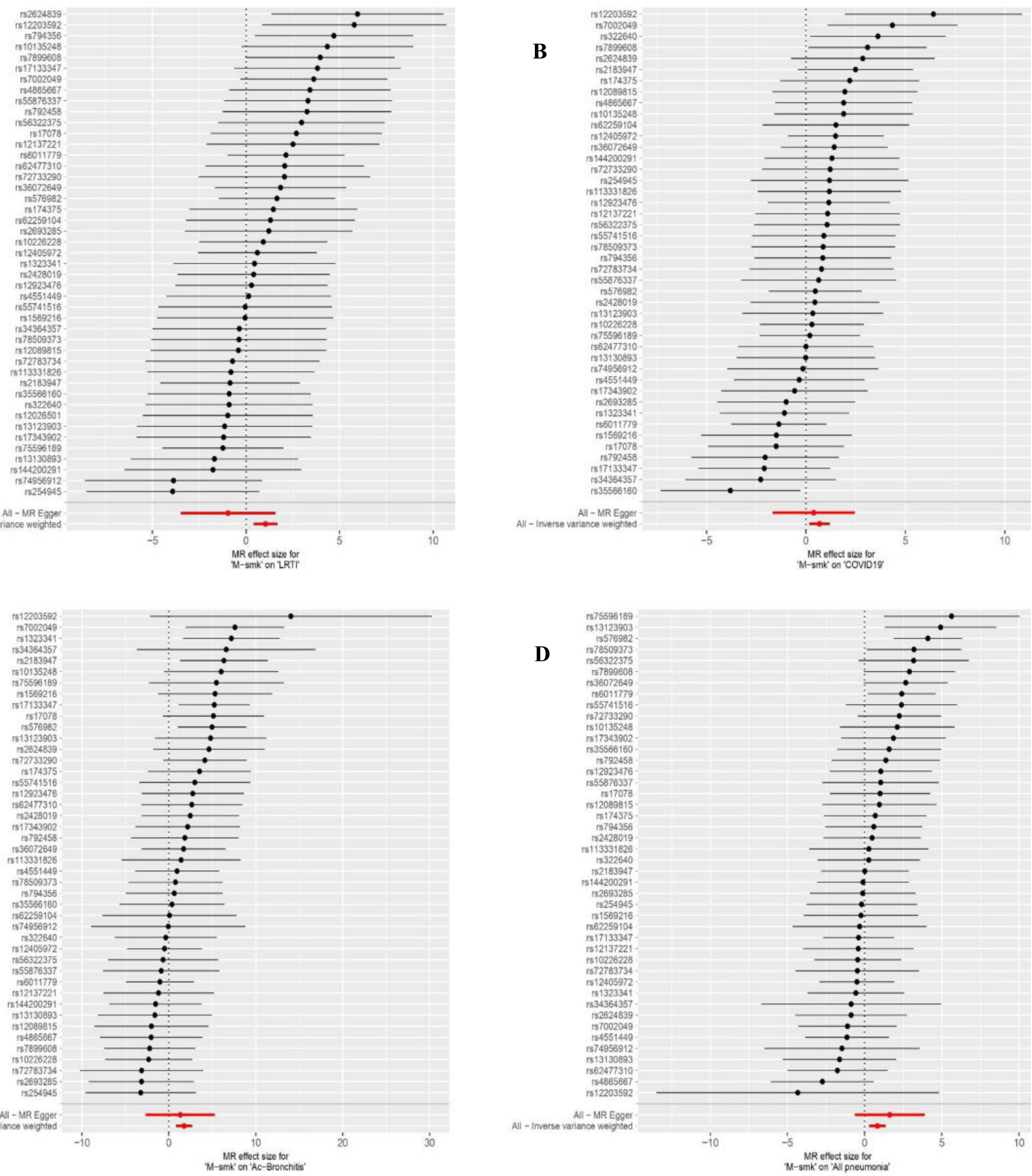
A
rs2624839
rs12203592
rs794356
rs10135248
rs7899608
rs17133347
rs7002049
rs4865667
rs55876337
rs792458
rs56322375
rs17078
rs12137221
rs6011779
rs62477310
rs72733290
rs36072649
rs576982
rs174375
rs62259104
rs2693285
rs10226228
rs12405972
rs1323341
rs2428019
rs12923476
rs4551449
rs55741516
rs1569216
rs34364357
rs78509373
rs12089815
rs72783734
rs113331826
rs2183947
rs35566160
rs322640
rs12026501
rs13123903
rs17343902
rs75596189
rs13130893
rs144200291
rs74956912
rs254945
All − MR Egger
All − Inverse variance weighted
-5
0
5
10
MR effect size for
'M−smk' on 'LRTI'
B
rs12203592
rs7002049
rs322640
rs7899608
rs2624839
rs2183947
rs174375
rs12089815
rs4865667
rs10135248
rs62259104
rs12405972
rs36072649
rs144200291
rs72733290
rs254945
rs113331826
rs12923476
rs12137221
rs56322375
rs55741516
rs78509373
rs794356
rs72783734
rs55876337
rs576982
rs2428019
rs13123903
rs10226228
rs75596189
rs62477310
rs13130893
rs74956912
rs4551449
rs17343902
rs2693285
rs1323341
rs6011779
rs1569216
rs17078
rs792458
rs17133347
rs34364357
rs35566160
All − MR Egger
All − Inverse variance weighted
-5
0
5
10
MR effect size for
'M−smk' on 'COVID19'
C
rs12203592
rs7002049
rs1323341
rs34364357
rs2183947
rs10135248
rs75596189
rs1569216
rs17133347
rs17078
rs576982
rs13123903
rs2624839
rs72733290
rs174375
rs55741516
rs12923476
rs62477310
rs2428019
rs17343902
rs792458
rs36072649
rs113331826
rs4551449
rs78509373
rs794356
rs35566160
rs62259104
rs74956912
rs322640
rs12405972
rs56322375
rs55876337
rs6011779
rs12137221
rs144200291
rs13130893
rs12089815
rs4865667
rs7899608
rs10226228
rs72783734
rs2693285
rs254945
All − MR Egger
All − Inverse variance weighted
-10
0
10
20
30
MR effect size for
'M−smk' on 'Ac−Bronchitis'
D
rs75596189
rs13123903
rs576982
rs78509373
rs56322375
rs7899608
rs36072649
rs6011779
rs55741516
rs72733290
rs10135248
rs17343902
rs35566160
rs792458
rs12923476
rs55876337
rs17078
rs12089815
rs174375
rs794356
rs2428019
rs113331826
rs322640
rs2183947
rs144200291
rs2693285
rs254945
rs1569216
rs62259104
rs17133347
rs12137221
rs10226228
rs72783734
rs12405972
rs1323341
rs34364357
rs2624839
rs7002049
rs4551449
rs74956912
rs13130893
rs62477310
rs4865667
rs12203592
All − MR Egger
All − Inverse variance weighted
-10
-5
0
5
10
MR effect size for
'M−smk' on 'All pneumonia'

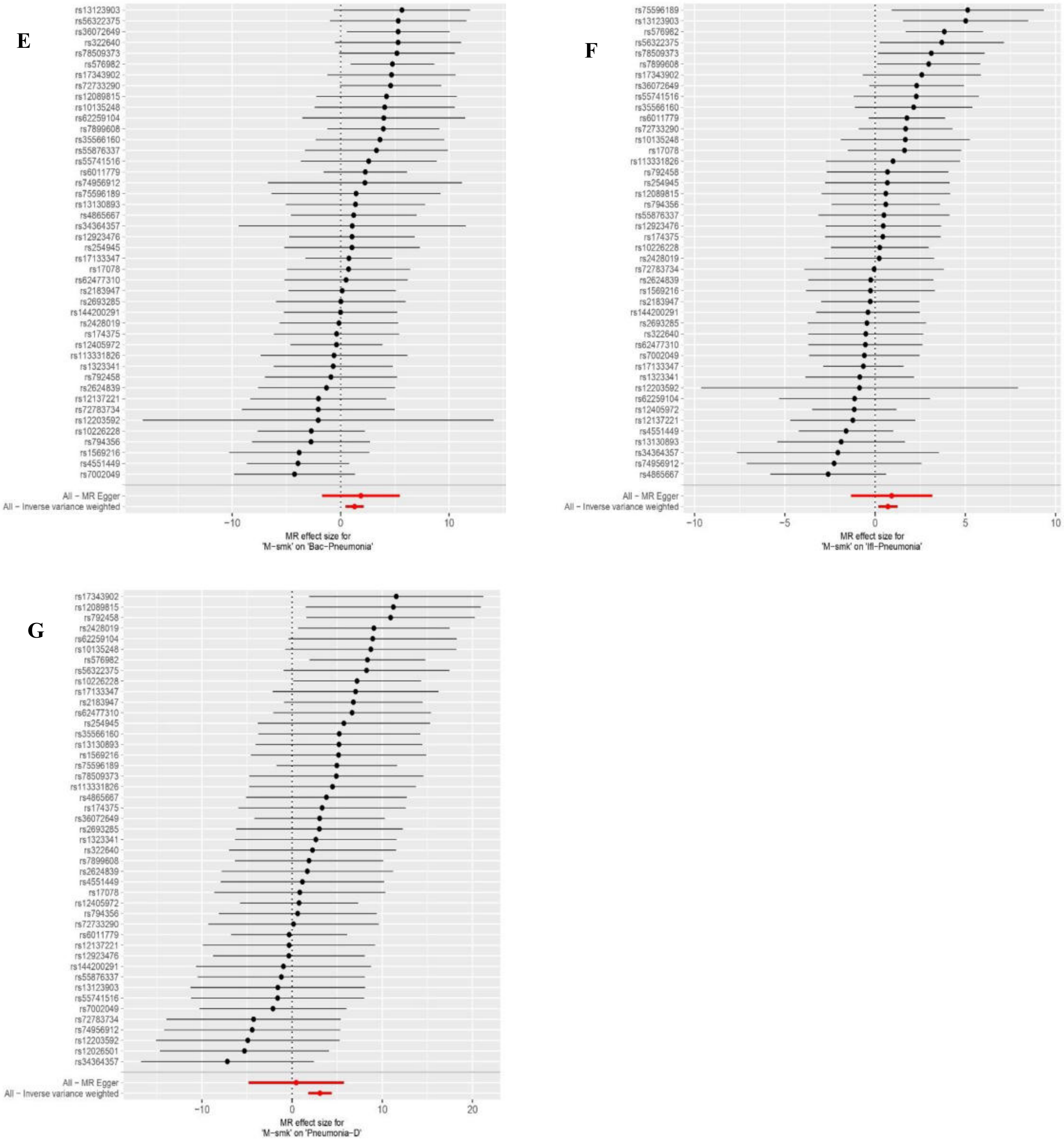

Figure S1. Forest plot of MR analysis before removing SNPs related to smoking by offspring.

The effect estimates for individual SNPs (black dots) and their corresponding 95% confidence intervals (horizontal lines) are presented. The pooled results from MR-Egger and Inverse variance weighted (IVW) analyses are shown as red dots and horizontal lines at the bottom. The IVW method indicated that maternal smoking around birth was associated with an increased risk of (A) LRTI (B) COVID-19, Coronavirus Disease 2019 (C) Acute bronchitis (D) Pneumonia (E) Bacterial pneumonia (F) Influenza pneumonia, and (G) Pneumonia death in adult offspring. This two-sample MR study utilized publicly available GWAS summary statistics (see Table 1 for detailed information). GWAS,

Genome-Wide Association Study; IVW, Inverse-Variance Weighted; LRTI, Lower Respiratory Tract Infection; MR, Mendelian Randomization; M-smk, Maternal smoking around birth; SNP, Single Nucleotide Polymorphism.

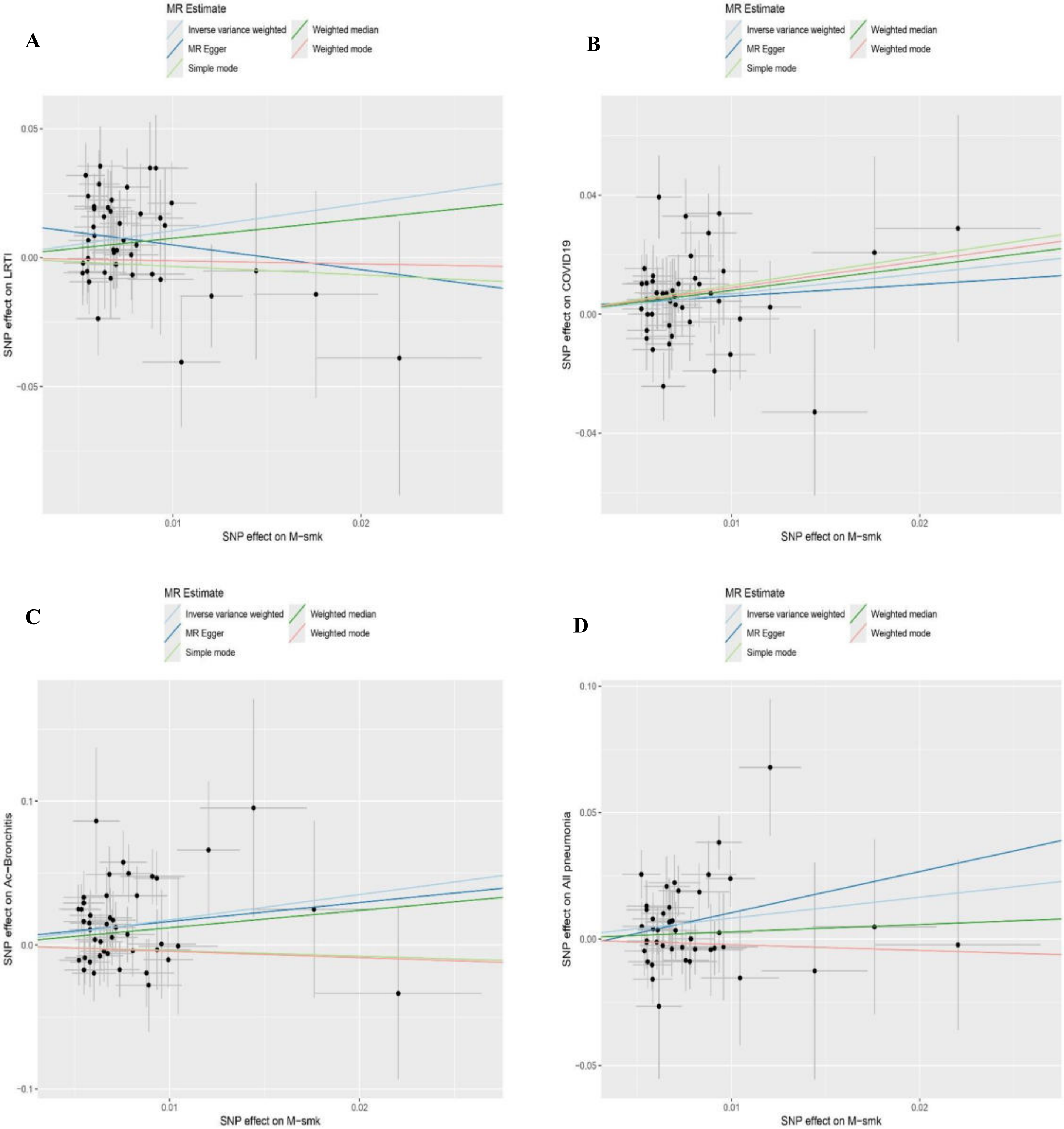

A
MR Estimate
Inverse variance weighted
MR Egger
Simple mode
Weighted median
Weighted mode
SNP effect on LRTI
SNP effect on M−smk
0.05
0.00
-0.05
0.01
0.02
B
MR Estimate
Inverse variance weighted
MR Egger
Simple mode
Weighted median
Weighted mode
SNP effect on COVID19
SNP effect on M−smk
0.04
0.00
-0.04
0.01
0.02
C
MR Estimate
Inverse variance weighted
MR Egger
Simple mode
Weighted median
Weighted mode
SNP effect on Ac−Bronchitis
SNP effect on M−smk
0.1
0.0
-0.1
0.01
0.02
D
MR Estimate
Inverse variance weighted
MR Egger
Simple mode
Weighted median
Weighted mode
SNP effect on All pneumonia
SNP effect on M−smk
0.10
0.05
0.00
-0.05
0.01
0.02

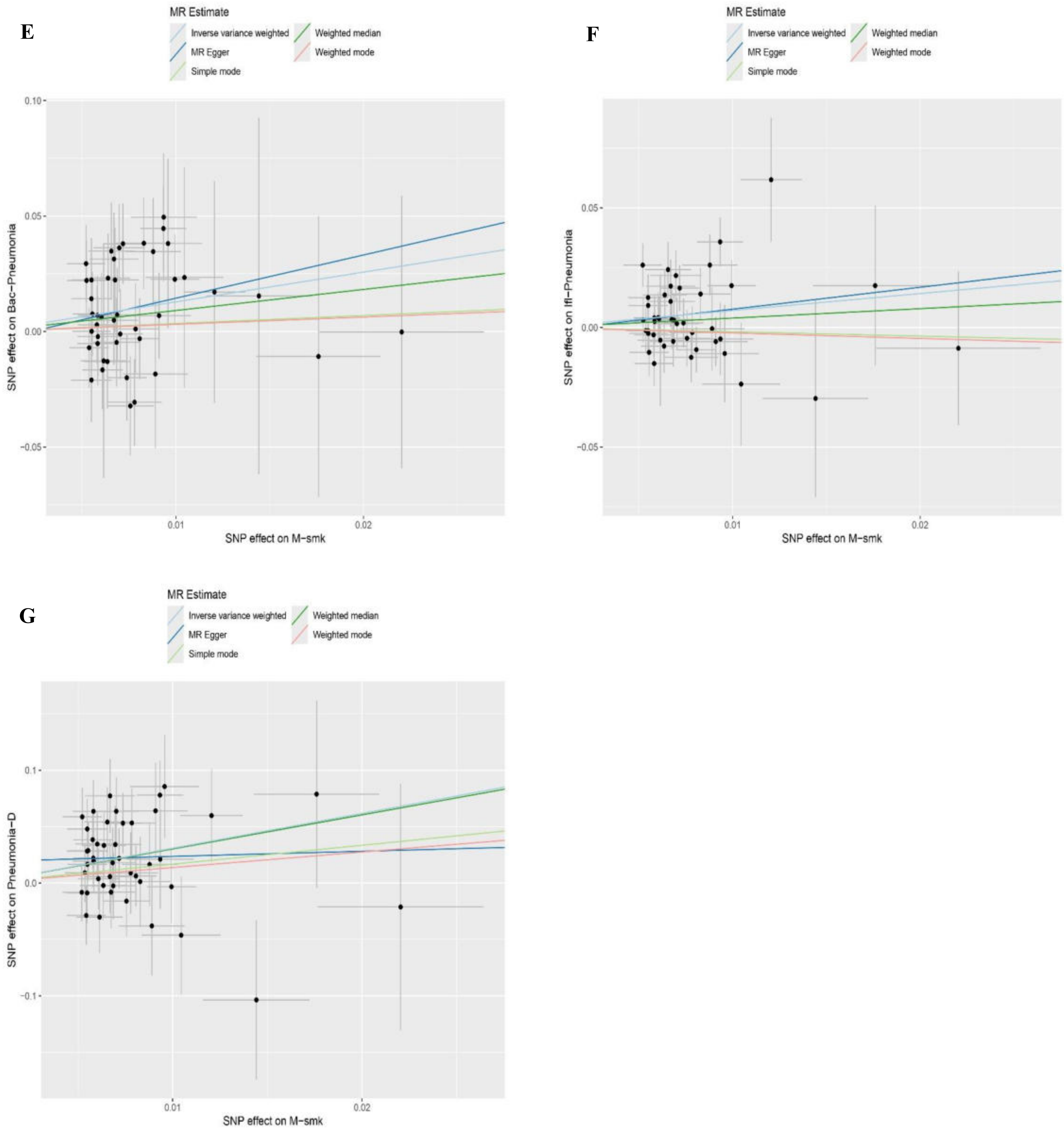

Figure S2. Scatter plot of MR analysis before removing SNPs related to smoking by offspring.

The plot displays the SNP effect sizes on the exposure (x-axis) against their effect sizes on the outcome (y-axis), with vertical and horizontal gray lines indicating 95% confidence intervals. The slopes of the colored lines represent the causal estimates derived from different MR methods, including Inverse variance weighted (IVW), MR-Egger, Simple mode, Weighted median, and Weighted mode. The IVW method (light blue line) indicated that maternal smoking around birth was associated with an increased risk of (A) LRTI (B) COVID-19, Coronavirus Disease 2019 (C) Acute bronchitis (D) Pneumonia (E) Bacterial pneumonia (F) Influenza pneumonia, and (G) Pneumonia death in adult

offspring. This two-sample MR study utilized publicly available GWAS summary statistics (see Table 1 for detailed information). GWAS, Genome-Wide Association Study; IVW, Inverse-Variance Weighted; LRTI, Lower Respiratory Tract Infection; MR, Mendelian Randomization; M-smk, Maternal smoking around birth; SNP, Single Nucleotide Polymorphism.

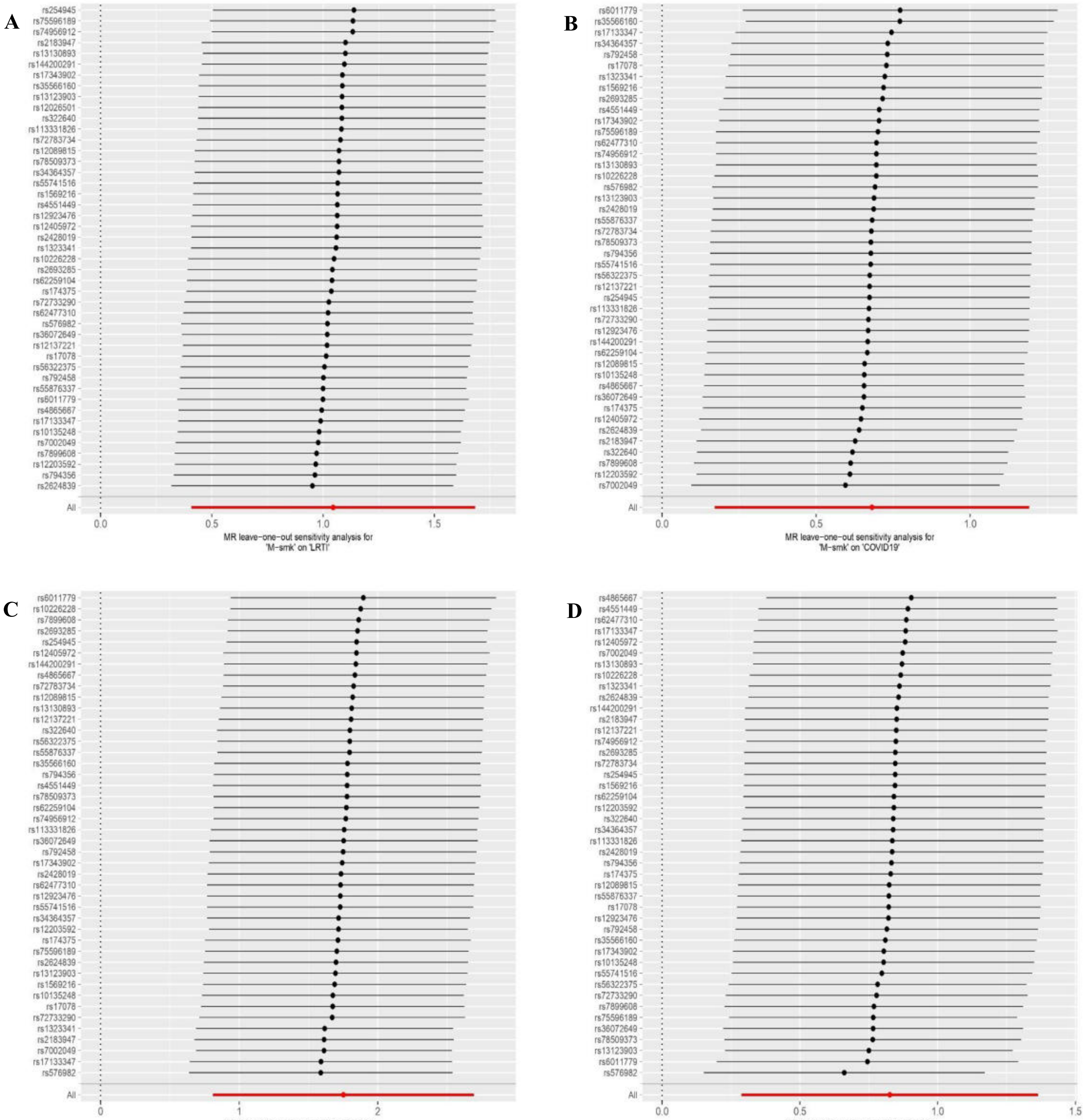
A
All
0.0
0.5
1.0
1.5
MR leave−one−out sensitivity analysis for
'M−smk' on 'LRTI'
B
All
0.0
0.5
1.0
MR leave−one−out sensitivity analysis for
'M−smk' on 'COVID19'
C
All
0
1
2
MR leave−one−out sensitivity analysis for
'M−smk' on 'Ac−Bronchitis'
D
All
0.0
0.5
1.0
1.5
MR leave−one−out sensitivity analysis for
'M−smk' on 'All pneumonia'

E F

G

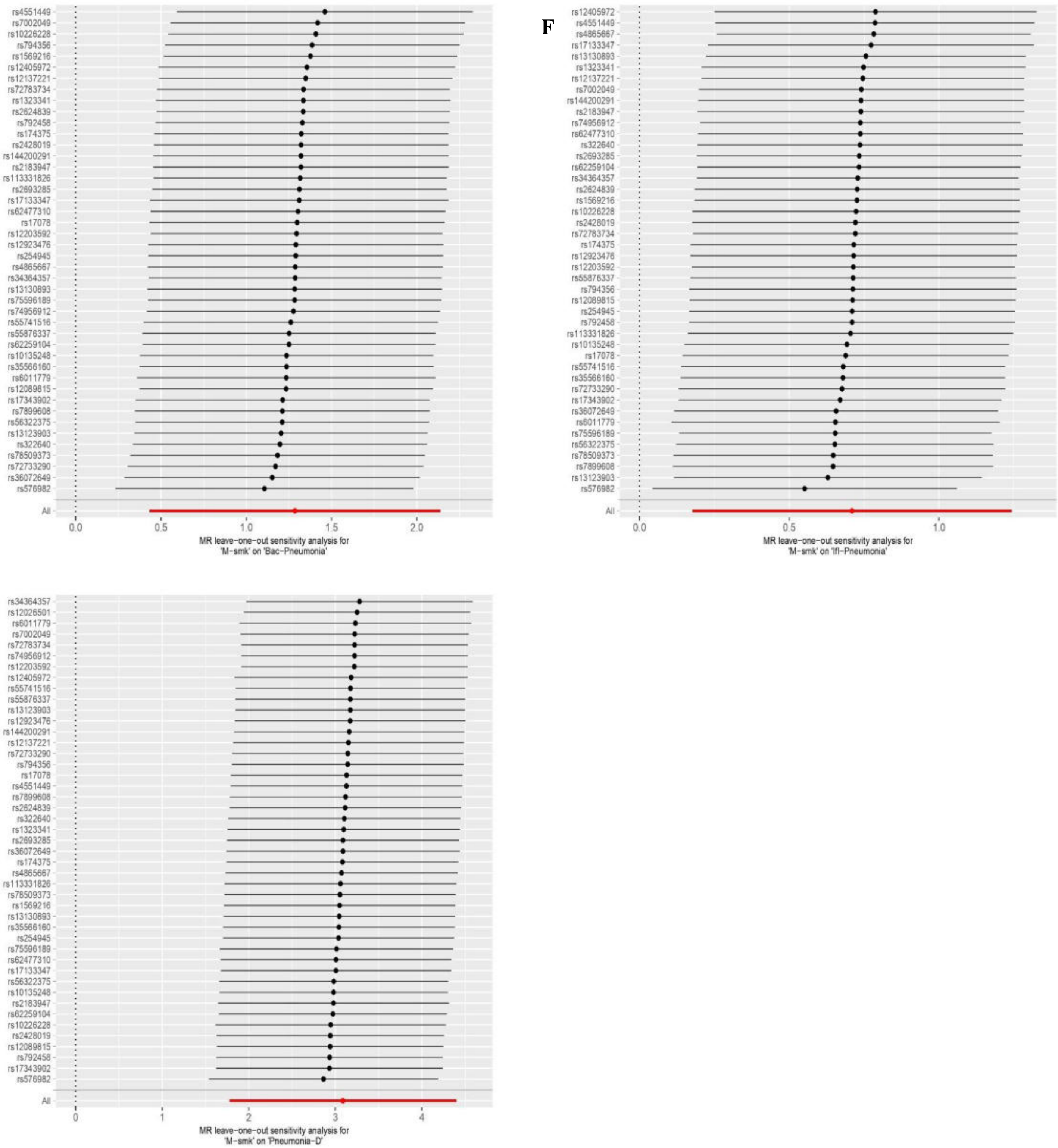

Figure S3. Leave-one-out sensitivity analysis before removing SNPs related to smoking by offspring.

Leave-one-out sensitivity analyses were performed to assess the associations of maternal smoking around birth with (A) LRTI, (B) COVID-19, coronavirus disease 2019, (C) acute bronchitis, (D) pneumonia, (E) bacterial pneumonia, (F) influenza pneumonia, and (G) pneumonia death in adult offspring. The plot displays the causal estimates (black dots) and their corresponding 95% confidence intervals (horizontal lines) obtained by sequentially removing each SNP from the analysis. The red dot and line at the bottom represent the pooled estimate using all SNPs. The leave-one-out analysis revealed that the estimates remained robust and stable, indicating that the observed associations (A-

G) were not driven by any single SNP. This two-sample MR study utilized publicly available GWAS summary statistics (see Table 1 for detailed information). GWAS, Genome-Wide Association Study; LRTI, Lower Respiratory Tract Infection; M-smk, Maternal smoking around birth; SNP, Single Nucleotide Polymorphism.

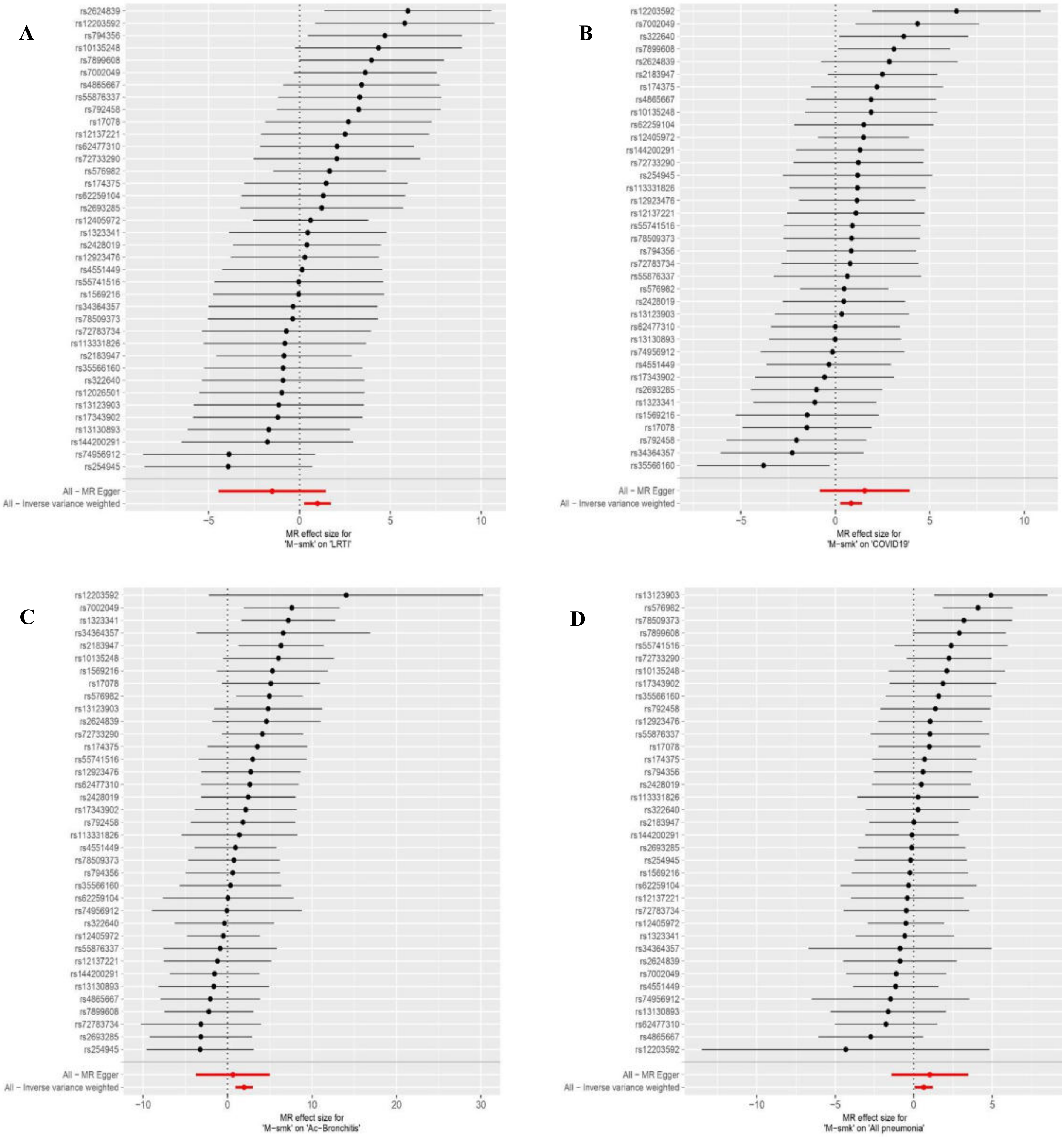
A
rs2624839
rs12203592
rs794356
rs10135248
rs7899608
rs7002049
rs4865667
rs55876337
rs792458
rs17078
rs12137221
rs62477310
rs72733290
rs576982
rs174375
rs62259104
rs2693285
rs12405972
rs1323341
rs2428019
rs12923476
rs4551449
rs55741516
rs1569216
rs34364357
rs78509373
rs72783734
rs113331826
rs2183947
rs35566160
rs322640
rs12026501
rs13123903
rs17343902
rs13130893
rs144200291
rs74956912
rs254945
All − MR Egger
All − Inverse variance weighted
-5
0
5
10
MR effect size for
'M−smk' on 'LRTI'
B
rs12203592
rs7002049
rs322640
rs7899608
rs2624839
rs2183947
rs174375
rs4865667
rs10135248
rs62259104
rs12405972
rs144200291
rs72733290
rs254945
rs113331826
rs12923476
rs12137221
rs55741516
rs78509373
rs794356
rs72783734
rs55876337
rs576982
rs2428019
rs13123903
rs62477310
rs13130893
rs74956912
rs4551449
rs17343902
rs2693285
rs1323341
rs1569216
rs17078
rs792458
rs34364357
rs35566160
All − MR Egger
All − Inverse variance weighted
-5
0
5
10
MR effect size for
'M−smk' on 'COVID19'
C
rs12203592
rs7002049
rs1323341
rs34364357
rs2183947
rs10135248
rs1569216
rs17078
rs576982
rs13123903
rs2624839
rs72733290
rs174375
rs55741516
rs12923476
rs62477310
rs2428019
rs17343902
rs792458
rs113331826
rs4551449
rs78509373
rs794356
rs35566160
rs62259104
rs74956912
rs322640
rs12405972
rs55876337
rs12137221
rs144200291
rs13130893
rs4865667
rs7899608
rs72783734
rs2693285
rs254945
All − MR Egger
All − Inverse variance weighted
-10
0
10
20
30
MR effect size for
'M−smk' on 'Ac−Bronchitis'
D
rs13123903
rs576982
rs78509373
rs7899608
rs55741516
rs72733290
rs10135248
rs17343902
rs35566160
rs792458
rs12923476
rs55876337
rs17078
rs174375
rs794356
rs2428019
rs113331826
rs322640
rs2183947
rs144200291
rs2693285
rs254945
rs1569216
rs62259104
rs12137221
rs72783734
rs12405972
rs1323341
rs34364357
rs2624839
rs7002049
rs4551449
rs74956912
rs13130893
rs62477310
rs4865667
rs12203592
All − MR Egger
All − Inverse variance weighted
-10
-5
0
5
MR effect size for
'M−smk' on 'All pneumonia'

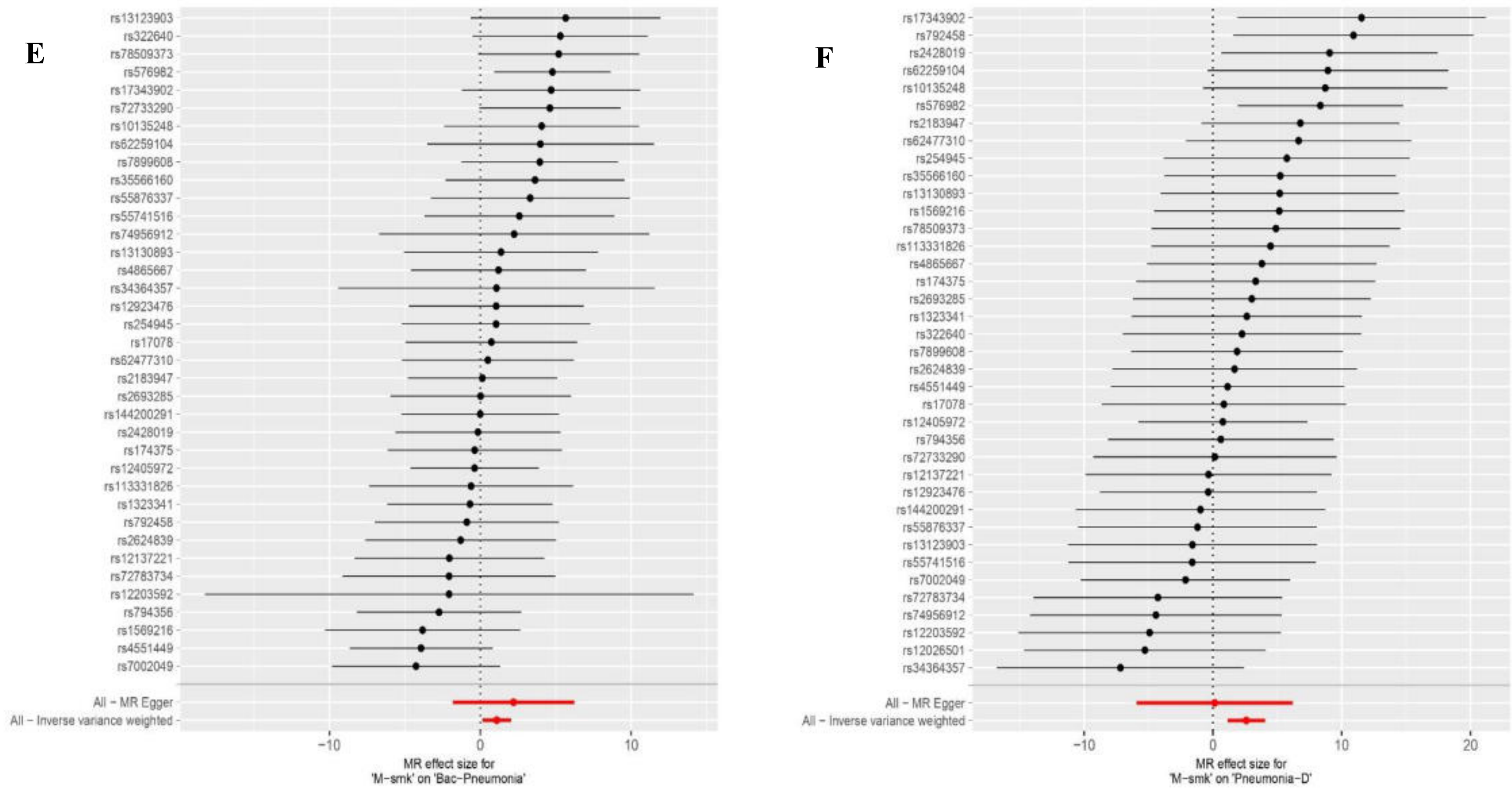

Figure S4. Forest plot of MR analysis after removing SNPs related to smoking by offspring.

The effect estimates for individual SNPs (black dots) and their corresponding 95% confidence intervals (horizontal lines) are presented. The pooled results from MR-Egger and Inverse variance weighted (IVW) analyses are shown as red dots and horizontal lines at the bottom. The IVW method indicated that maternal smoking around birth was associated with an increased risk of (A) LRTI (B) COVID-19, Coronavirus Disease 2019 (C) Acute bronchitis (D) Pneumonia (E) Bacterial pneumonia, and (F) Pneumonia death in adult offspring. This two-sample MR study utilized publicly available GWAS summary statistics (see Table 1 for detailed information). GWAS, Genome-Wide Association Study; IVW, Inverse-Variance Weighted; LRTI, Lower Respiratory Tract Infection; MR, Mendelian Randomization; M-smk, Maternal smoking around birth; SNP, Single Nucleotide Polymorphism.

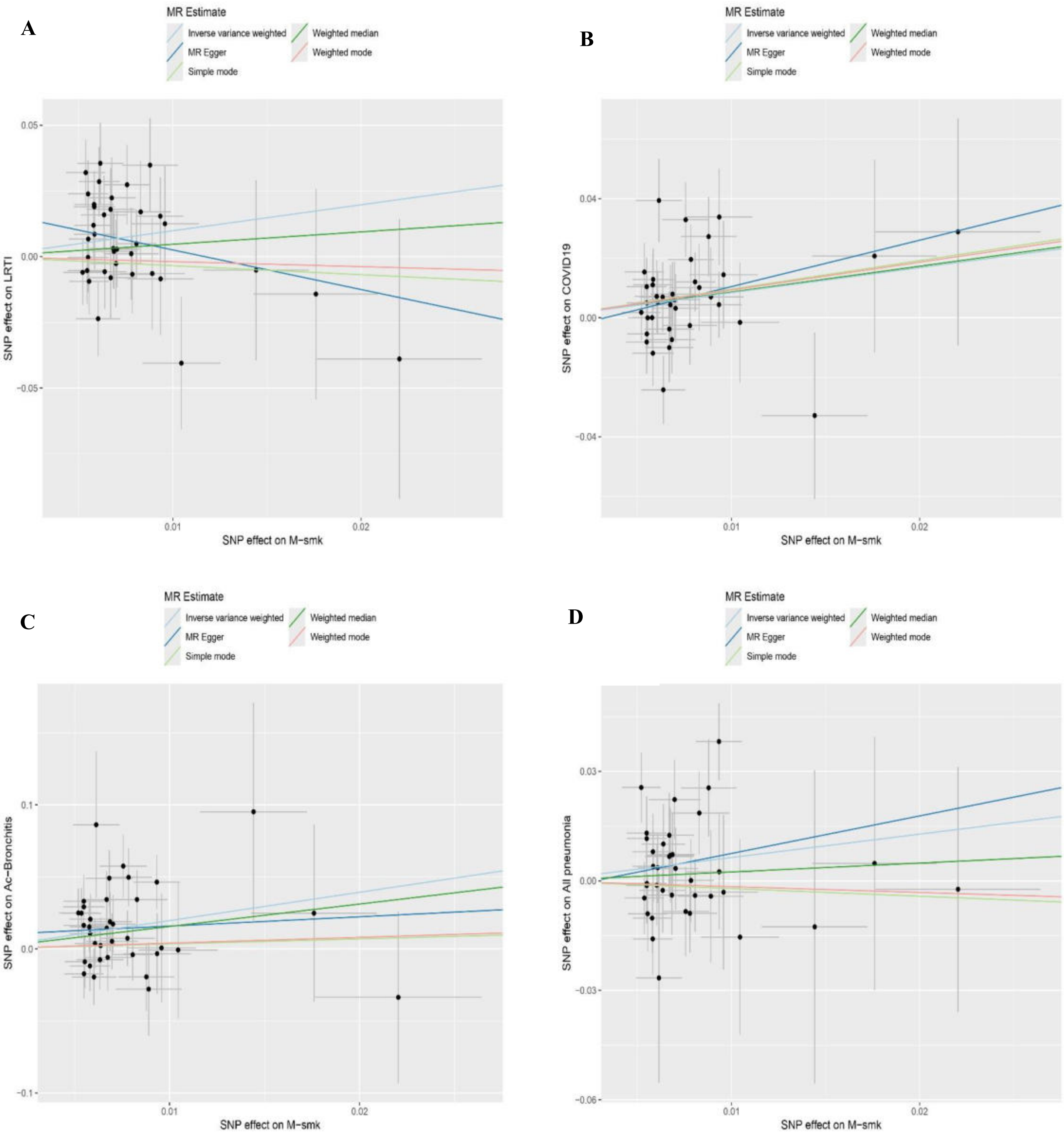
A
MR Estimate
Inverse variance weighted
MR Egger
Simple mode
Weighted median
Weighted mode
SNP effect on LRTI
0.05
0.00
-0.05
SNP effect on M−smk
0.01
0.02
B
MR Estimate
Inverse variance weighted
MR Egger
Simple mode
Weighted median
Weighted mode
SNP effect on COVID19
0.04
0.00
-0.04
SNP effect on M−smk
0.01
0.02
C
MR Estimate
Inverse variance weighted
MR Egger
Simple mode
Weighted median
Weighted mode
SNP effect on Ac−Bronchitis
0.1
0.0
-0.1
SNP effect on M−smk
0.01
0.02
D
MR Estimate
Inverse variance weighted
MR Egger
Simple mode
Weighted median
Weighted mode
SNP effect on All pneumonia
0.03
0.00
-0.03
-0.06
SNP effect on M−smk
0.01
0.02

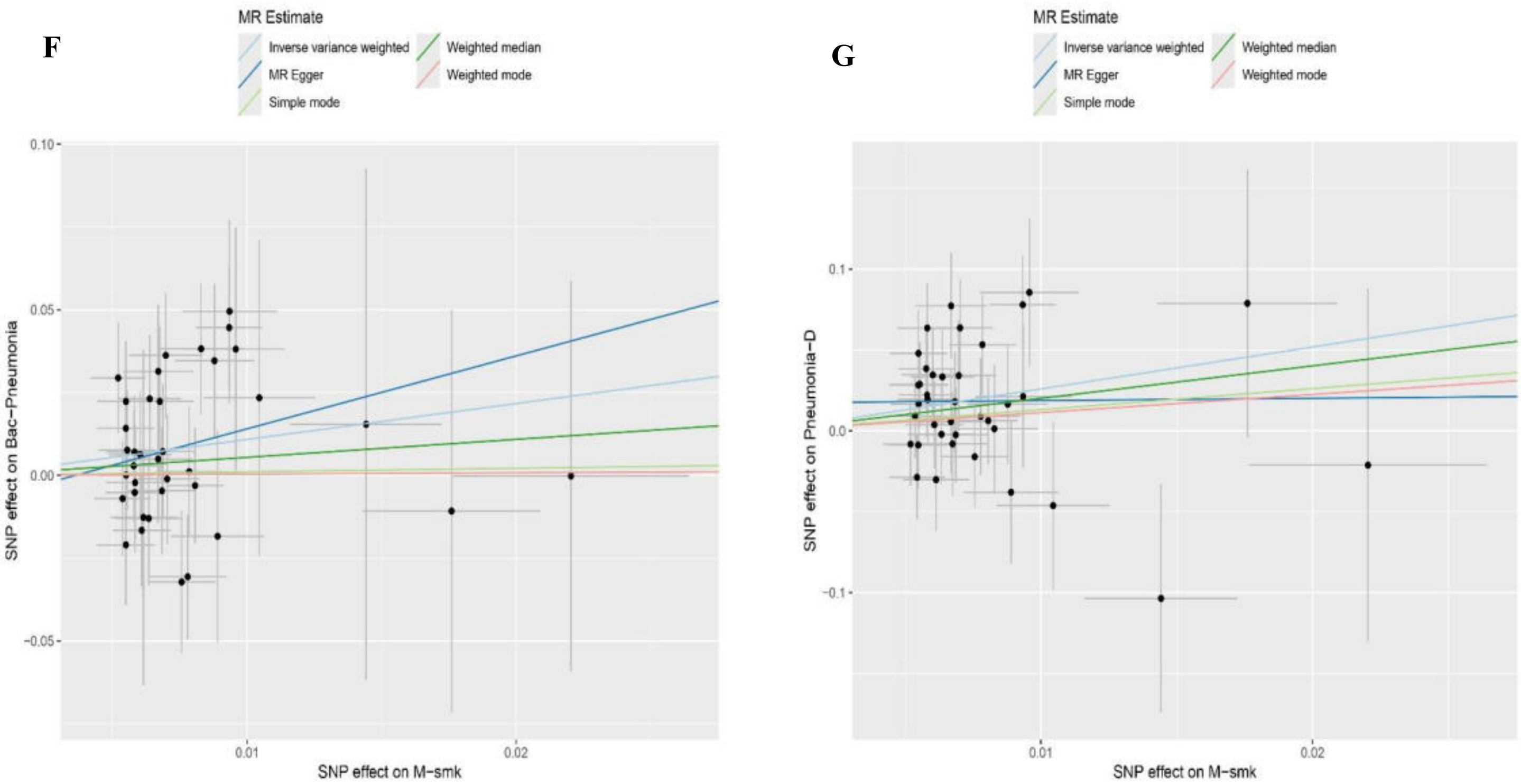

Figure S5. Scatter plot of MR analysis after removing SNPs related to smoking by offspring.

The plot displays the SNP effect sizes on the exposure (x-axis) against their effect sizes on the outcome (y-axis), with vertical and horizontal gray lines indicating 95% confidence intervals. The slopes of the colored lines represent the causal estimates derived from different MR methods, including Inverse variance weighted (IVW), MR-Egger, Simple mode, Weighted median, and Weighted mode. The IVW method (light blue line) indicated that maternal smoking around birth was associated with an increased risk of (A) LRTI (B) COVID-19, Coronavirus Disease 2019 (C) Acute bronchitis (D) Pneumonia (E) Bacterial pneumonia, and (F) Pneumonia death in adult offspring. This two-sample MR study utilized publicly available GWAS summary statistics (see Table 1 for detailed information). GWAS, Genome-Wide Association Study; IVW, Inverse-Variance Weighted; LRTI, Lower Respiratory Tract Infection; MR, Mendelian Randomization; M-smk, Maternal smoking around birth; SNP, Single Nucleotide Polymorphism.

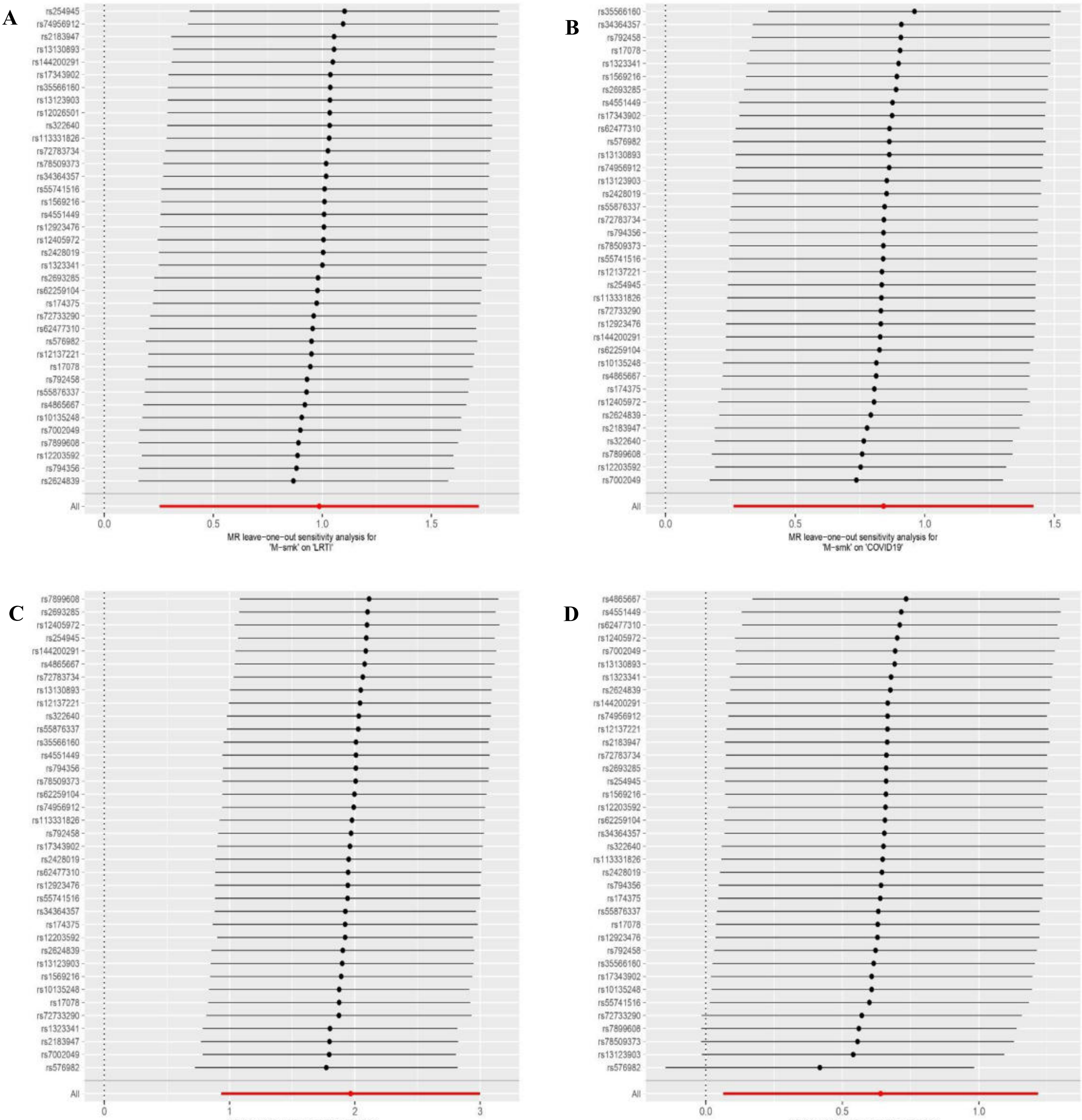
A
rs254945
rs74956912
rs2183947
rs13130893
rs144200291
rs17343902
rs35566160
rs13123903
rs12026501
rs322640
rs113331826
rs72783734
rs78509373
rs34364357
rs55741516
rs1569216
rs4551449
rs12923476
rs12405972
rs2428019
rs1323341
rs2693285
rs62259104
rs174375
rs72733290
rs62477310
rs576982
rs12137221
rs17078
rs792458
rs55876337
rs4865667
rs10135248
rs7002049
rs7899608
rs12203592
rs794356
rs2624839
All
0.0
0.5
1.0
1.5
MR leave-one-out sensitivity analysis for
'M-smk' on 'LRTI'
B
rs35566160
rs34364357
rs792458
rs17078
rs1323341
rs1569216
rs2693285
rs4551449
rs17343902
rs62477310
rs576982
rs13130893
rs74956912
rs13123903
rs2428019
rs55876337
rs72783734
rs794356
rs78509373
rs55741516
rs12137221
rs254945
rs113331826
rs72733290
rs12923476
rs144200291
rs62259104
rs10135248
rs4865667
rs174375
rs12405972
rs2624839
rs2183947
rs322640
rs7899608
rs12203592
rs7002049
All
0.0
0.5
1.0
1.5
MR leave-one-out sensitivity analysis for
'M-smk' on 'COVID19'
C
rs7899608
rs2693285
rs12405972
rs254945
rs144200291
rs4865667
rs72783734
rs13130893
rs12137221
rs322640
rs55876337
rs35566160
rs4551449
rs794356
rs78509373
rs62259104
rs74956912
rs113331826
rs792458
rs17343902
rs2428019
rs62477310
rs12923476
rs55741516
rs34364357
rs174375
rs12203592
rs2624839
rs13123903
rs1569216
rs10135248
rs17078
rs72733290
rs1323341
rs2183947
rs7002049
rs576982
All
0
1
2
3
MR leave-one-out sensitivity analysis for
'M-smk' on 'Ac-Bronchitis'
D
rs4865667
rs4551449
rs62477310
rs12405972
rs7002049
rs13130893
rs1323341
rs2624839
rs144200291
rs74956912
rs12137221
rs2183947
rs72783734
rs2693285
rs254945
rs1569216
rs12203592
rs62259104
rs34364357
rs322640
rs113331826
rs2428019
rs794356
rs174375
rs55876337
rs17078
rs12923476
rs792458
rs35566160
rs17343902
rs10135248
rs55741516
rs72733290
rs7899608
rs78509373
rs13123903
rs576982
All
0.0
0.5
1.0
MR leave-one-out sensitivity analysis for
'M-smk' on 'All pneumonia'

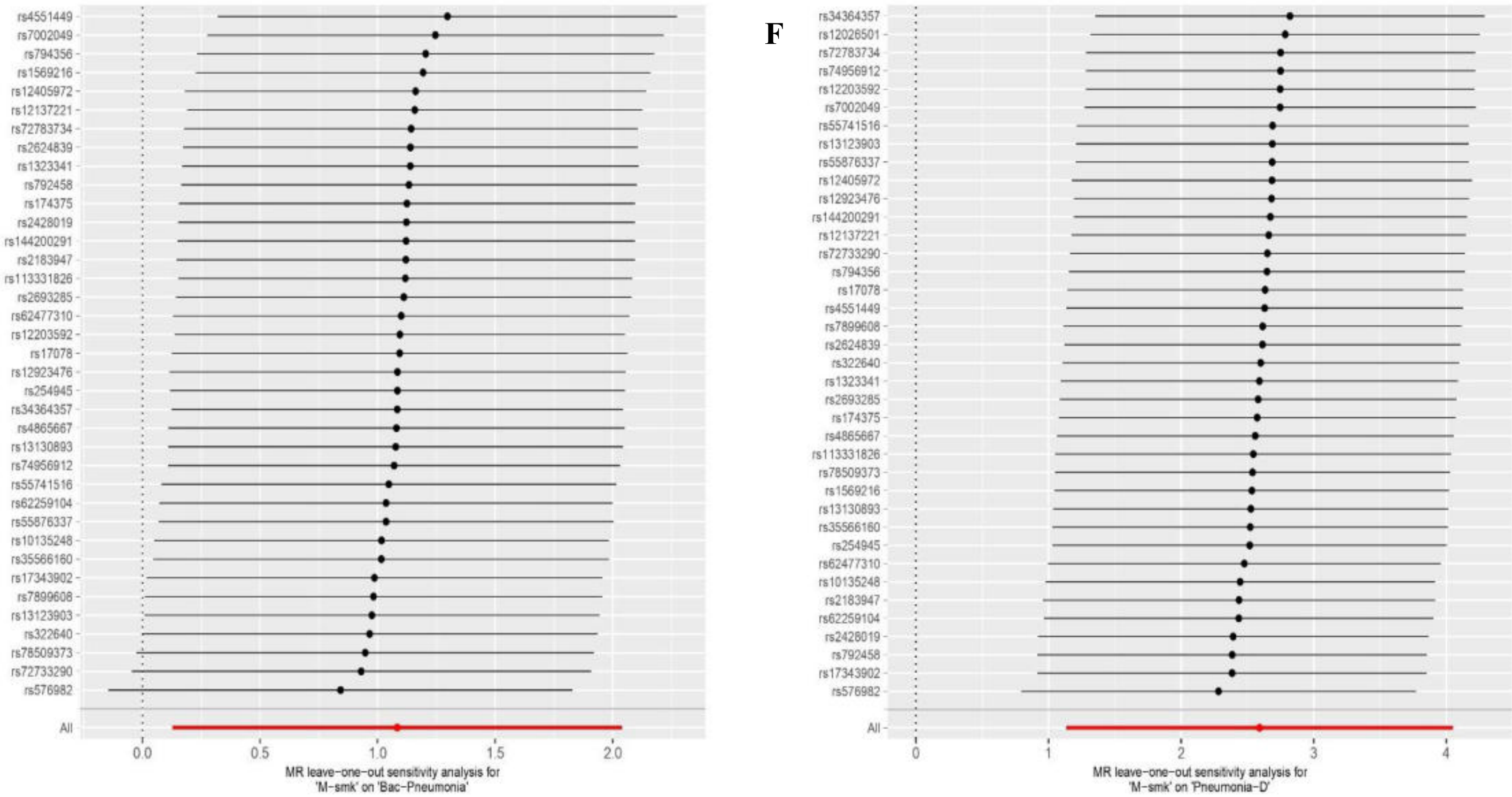

Figure S6. Leave-one-out sensitivity analysis after removing SNPs related to smoking by offspring.

Leave-one-out sensitivity analyses were performed to assess the associations of maternal smoking around birth with (A) LRTI, (B) COVID-19 (coronavirus disease 2019), (C) acute bronchitis, (D) pneumonia, (E) bacterial pneumonia, and (F) pneumonia death in adult offspring. The plot displays the causal estimates (black dots) and their corresponding 95% confidence intervals (horizontal lines) obtained by sequentially removing each SNP from the analysis. The red dot and line at the bottom represent the pooled estimate using all SNPs. The leave-one-out analysis revealed that the estimates remained robust and stable, indicating that the observed associations (A-F) were not driven by any single SNP. This two-sample MR study utilized publicly available GWAS summary statistics (see Table 1 for detailed information). GWAS, Genome-Wide Association Study; LRTI, Lower Respiratory Tract Infection; M-smk, Maternal smoking around birth; SNP, Single Nucleotide Polymorphism.

Table S1. Characteristics of the genetic variants associated with maternal smoking around birth and URTI.

SNP	Chr.	Position	Effect allele	Other allele	ß_exp	ß_out	SE_exp	Pvalue_exp	SE_out	Pvalue_out	Fvalue
rs10135248	14	104122310	T	C	-0.005	0.006	0.001	2.20E-07	0.029	0.828	26.848
rs10226228	7	32315613	A	G	0.007	0.012	0.001	4.00E-12	0.029	0.679	48.137
rs113331826	11	29861212	T	C	-0.018	-0.035	0.003	1.10E-07	0.090	0.694	28.271
rs12026501	1	237995526	T	A	-0.005	0.031	0.001	1.60E-07	0.028	0.271	27.498
rs12089815	1	91189933	G	A	-0.005	-0.004	0.001	4.10E-07	0.028	0.890	25.668
rs12137221	1	50182298	C	T	0.006	0.052	0.001	2.70E-07	0.034	0.123	26.473
rs12203592	6	396321	C	T	0.006	0.043	0.001	4.80E-07	0.035	0.214	25.335
rs12405972	1	44097438	G	T	-0.008	-0.022	0.001	6.40E-14	0.029	0.447	56.247
rs12923476	16	24798079	G	A	-0.007	0.022	0.001	4.80E-09	0.032	0.487	34.260
rs13123903	4	35513601	C	T	0.005	0.012	0.001	3.80E-07	0.028	0.677	25.814
rs13130893	4	46983522	A	G	0.006	-0.012	0.001	1.00E-07	0.028	0.671	28.327
rs1323341	9	14453010	A	G	-0.007	0.012	0.001	3.90E-08	0.034	0.733	30.203
rs144200291	3	51921338	G	A	-0.022	-0.081	0.004	5.00E-07	0.119	0.494	25.270
rs1569216	4	172473450	A	G	0.005	-0.029	0.001	4.40E-07	0.030	0.331	25.520
rs17078	10	106653311	A	G	-0.007	-0.058	0.001	2.00E-07	0.035	0.098	27.028
rs17133347	7	3472721	C	T	-0.009	-0.005	0.002	9.00E-08	0.046	0.917	28.577
rs17343902	5	103677751	A	G	-0.007	-0.024	0.001	3.30E-07	0.036	0.500	26.053
rs174375	6	111908266	C	G	-0.006	-0.075	0.001	1.20E-07	0.030	0.013	27.986
rs2183947	6	26159356	G	A	-0.008	0.007	0.001	1.50E-10	0.033	0.831	40.977
rs2428019	11	113678423	C	A	0.007	-0.031	0.001	5.10E-09	0.033	0.339	34.158
rs254945	16	8956703	G	A	-0.006	-0.028	0.001	2.60E-07	0.032	0.380	26.498
rs2624839	3	50202231	T	C	0.005	0.034	0.001	2.30E-07	0.028	0.228	26.790
rs2693285	2	199702908	A	G	0.005	-0.018	0.001	1.00E-07	0.028	0.517	28.323
rs322640	18	34592625	A	T	-0.009	-0.044	0.002	1.10E-07	0.048	0.359	28.271
rs34364357	3	140346921	A	T	-0.014	0.058	0.003	2.90E-07	0.076	0.447	26.291
rs35566160	2	164928199	A	G	0.006	-0.013	0.001	4.50E-08	0.032	0.680	29.927
rs36072649	4	140939110	T	A	-0.007	-0.012	0.001	1.10E-11	0.029	0.689	46.094
rs4551449	9	22610366	C	T	-0.008	-0.009	0.001	6.80E-08	0.039	0.829	29.116
rs4865667	5	50748173	C	T	-0.006	-0.031	0.001	3.40E-08	0.029	0.281	30.451
rs55741516	14	42697579	T	C	-0.005	0.014	0.001	2.90E-07	0.029	0.623	26.309
rs55876337	2	137399539	C	A	0.007	-0.020	0.001	1.10E-07	0.035	0.572	28.276
rs56322375	17	32906373	G	T	0.007	-0.038	0.001	9.40E-08	0.033	0.249	28.493
rs576982	15	78870803	C	T	-0.009	0.016	0.001	2.30E-14	0.033	0.626	58.275
rs6011779	20	61984317	C	T	-0.010	-0.011	0.001	2.50E-14	0.035	0.754	58.089
rs62259104	3	67116763	T	C	0.010	-0.019	0.002	1.40E-07	0.050	0.708	27.694
rs62477310	7	114951541	T	C	-0.006	-0.003	0.001	2.00E-08	0.028	0.901	31.496
rs7002049	8	93114414	T	C	0.008	0.065	0.001	1.40E-09	0.034	0.057	36.620
rs72733290	1	193723842	G	A	-0.008	0.011	0.002	2.10E-07	0.043	0.798	26.932
rs72783734	5	86811301	C	A	-0.009	-0.087	0.002	3.10E-07	0.047	0.066	26.163
rs74956912	2	162878335	G	A	-0.010	0.077	0.002	5.00E-07	0.056	0.173	25.256
rs75596189	9	136468701	C	T	0.012	0.057	0.002	2.10E-13	0.045	0.199	53.864
rs78509373	17	43266542	T	C	0.007	-0.087	0.001	3.50E-07	0.037	0.019	25.936
rs7899608	10	104727304	C	T	0.009	0.104	0.001	2.30E-09	0.040	0.010	35.667
rs792458	10	22133793	T	G	-0.006	-0.053	0.001	1.20E-07	0.030	0.079	27.957
rs794356	7	75196531	G	A	-0.006	-0.041	0.001	1.90E-08	0.029	0.167	31.546

Chr., Chromosome; exp, exposure; out, outcome, SE, Standard Error; SNP, Single Nucleotide Polymorphism; URTI, Upper Respiratory Tract

Infection.

Table S2. Characteristics of the genetic variants associated with maternal smoking around birth and LRTI.

SNP	Chr.	Position	Effect allele	Other allele	ß_exp	ß_out	SE_exp	Pvalue_exp	SE_out	Pvalue_out	Fvalue
rs10135248	14	104122310	T	C	-0.005	-0.024	0.001	2.20E-07	0.013	0.064	26.848
rs10226228	7	32315613	A	G	0.007	0.007	0.001	4.00E-12	0.013	0.598	48.137
rs113331826	11	29861212	T	C	-0.018	0.014	0.003	1.10E-07	0.040	0.723	28.271
rs12026501	1	237995526	T	A	-0.005	0.005	0.001	1.60E-07	0.013	0.674	27.498
rs12089815	1	91189933	G	A	-0.005	0.002	0.001	4.10E-07	0.013	0.865	25.668
rs12137221	1	50182298	C	T	0.006	0.016	0.001	2.70E-07	0.015	0.288	26.473
rs12203592	6	396321	C	T	0.006	0.036	0.001	4.80E-07	0.015	0.021	25.335
rs12405972	1	44097438	G	T	-0.008	-0.005	0.001	6.40E-14	0.013	0.707	56.247
rs12923476	16	24798079	G	A	-0.007	-0.002	0.001	4.80E-09	0.014	0.888	34.260
rs13123903	4	35513601	C	T	0.005	-0.006	0.001	3.80E-07	0.012	0.632	25.814
rs13130893	4	46983522	A	G	0.006	-0.009	0.001	1.00E-07	0.013	0.459	28.327
rs1323341	9	14453010	A	G	-0.007	-0.003	0.001	3.90E-08	0.015	0.838	30.203
rs144200291	3	51921338	G	A	-0.022	0.039	0.004	5.00E-07	0.053	0.464	25.270
rs1569216	4	172473450	A	G	0.005	0.000	0.001	4.40E-07	0.013	0.982	25.520
rs17078	10	106653311	A	G	-0.007	-0.018	0.001	2.00E-07	0.016	0.250	27.028
rs17133347	7	3472721	C	T	-0.009	-0.035	0.002	9.00E-08	0.021	0.093	28.577
rs17343902	5	103677751	A	G	-0.007	0.008	0.001	3.30E-07	0.016	0.614	26.053
rs174375	6	111908266	C	G	-0.006	-0.009	0.001	1.20E-07	0.013	0.524	27.986
rs2183947	6	26159356	G	A	-0.008	0.007	0.001	1.50E-10	0.015	0.653	40.977
rs2428019	11	113678423	C	A	0.007	0.003	0.001	5.10E-09	0.015	0.845	34.158
rs254945	16	8956703	G	A	-0.006	0.024	0.001	2.60E-07	0.014	0.097	26.498
rs2624839	3	50202231	T	C	0.005	0.032	0.001	2.30E-07	0.013	0.011	26.790
rs2693285	2	199702908	A	G	0.005	0.007	0.001	1.00E-07	0.013	0.594	28.323
rs322640	18	34592625	A	T	-0.009	0.008	0.002	1.10E-07	0.021	0.692	28.271
rs34364357	3	140346921	A	T	-0.014	0.005	0.003	2.90E-07	0.034	0.881	26.291
rs35566160	2	164928199	A	G	0.006	-0.006	0.001	4.50E-08	0.014	0.686	29.927
rs36072649	4	140939110	T	A	-0.007	-0.013	0.001	1.10E-11	0.013	0.302	46.094
rs4551449	9	22610366	C	T	-0.008	-0.001	0.001	6.80E-08	0.017	0.949	29.116
rs4865667	5	50748173	C	T	-0.006	-0.020	0.001	3.40E-08	0.013	0.121	30.451
rs55741516	14	42697579	T	C	-0.005	0.000	0.001	2.90E-07	0.013	0.984	26.309
rs55876337	2	137399539	C	A	0.007	0.022	0.001	1.10E-07	0.015	0.148	28.276
rs56322375	17	32906373	G	T	0.007	0.019	0.001	9.40E-08	0.015	0.191	28.493
rs576982	15	78870803	C	T	-0.009	-0.015	0.001	2.30E-14	0.015	0.299	58.275
rs6011779	20	61984317	C	T	-0.010	-0.021	0.001	2.50E-14	0.016	0.181	58.089
rs62259104	3	67116763	T	C	0.010	0.013	0.002	1.40E-07	0.022	0.570	27.694
rs62477310	7	114951541	T	C	-0.006	-0.012	0.001	2.00E-08	0.013	0.340	31.496
rs7002049	8	93114414	T	C	0.008	0.027	0.001	1.40E-09	0.015	0.071	36.620
rs72733290	1	193723842	G	A	-0.008	-0.017	0.002	2.10E-07	0.019	0.380	26.932
rs72783734	5	86811301	C	A	-0.009	0.006	0.002	3.10E-07	0.021	0.762	26.163
rs74956912	2	162878335	G	A	-0.010	0.041	0.002	5.00E-07	0.025	0.109	25.256
rs75596189	9	136468701	C	T	0.012	-0.015	0.002	2.10E-13	0.020	0.455	53.864
rs78509373	17	43266542	T	C	0.007	-0.003	0.001	3.50E-07	0.017	0.874	25.936
rs7899608	10	104727304	C	T	0.009	0.035	0.001	2.30E-09	0.018	0.051	35.667
rs792458	10	22133793	T	G	-0.006	-0.019	0.001	1.20E-07	0.013	0.157	27.957
rs794356	7	75196531	G	A	-0.006	-0.029	0.001	1.90E-08	0.013	0.030	31.546

Chr., Chromosome; exp, exposure; out, outcome, SE, Standard Error; SNP, Single Nucleotide Polymorphism; LRTI, Lower Respiratory Tract

Infection.

Table S3. Characteristics of the genetic variants associated with maternal smoking around birth and pneumonia death.

SNP	Chr.	Position	Effect allele	Other allele	ß_exp	ß_out	SE_exp	Pvalue_exp	SE_out	Pvalue_out	Fvalue
rs10135248	14	104122310	T	C	-0.005	-0.048	0.001	2.20E-07	0.027	0.072	26.848
rs10226228	7	32315613	A	G	0.007	0.053	0.001	4.00E-12	0.027	0.047	48.137
rs113331826	11	29861212	T	C	-0.018	-0.079	0.003	1.10E-07	0.083	0.343	28.271
rs12026501	1	237995526	T	A	-0.005	0.029	0.001	1.60E-07	0.026	0.270	27.498
rs12089815	1	91189933	G	A	-0.005	-0.059	0.001	4.10E-07	0.026	0.024	25.668
rs12137221	1	50182298	C	T	0.006	-0.002	0.001	2.70E-07	0.031	0.945	26.473
rs12203592	6	396321	C	T	0.006	-0.030	0.001	4.80E-07	0.032	0.345	25.335
rs12405972	1	44097438	G	T	-0.008	-0.006	0.001	6.40E-14	0.027	0.817	56.247
rs12923476	16	24798079	G	A	-0.007	0.002	0.001	4.80E-09	0.029	0.933	34.260
rs13123903	4	35513601	C	T	0.005	-0.008	0.001	3.80E-07	0.026	0.748	25.814
rs13130893	4	46983522	A	G	0.006	0.029	0.001	1.00E-07	0.026	0.271	28.327
rs1323341	9	14453010	A	G	-0.007	-0.018	0.001	3.90E-08	0.031	0.564	30.203
rs144200291	3	51921338	G	A	-0.022	0.021	0.004	5.00E-07	0.109	0.846	25.270
rs1569216	4	172473450	A	G	0.005	0.028	0.001	4.40E-07	0.027	0.300	25.520
rs17078	10	106653311	A	G	-0.007	-0.006	0.001	2.00E-07	0.032	0.860	27.028
rs17133347	7	3472721	C	T	-0.009	-0.064	0.002	9.00E-08	0.043	0.133	28.577
rs17343902	5	103677751	A	G	-0.007	-0.077	0.001	3.30E-07	0.033	0.019	26.053
rs174375	6	111908266	C	G	-0.006	-0.019	0.001	1.20E-07	0.028	0.483	27.986
rs2183947	6	26159356	G	A	-0.008	-0.053	0.001	1.50E-10	0.031	0.083	40.977
rs2428019	11	113678423	C	A	0.007	0.064	0.001	5.10E-09	0.030	0.035	34.158
rs254945	16	8956703	G	A	-0.006	-0.035	0.001	2.60E-07	0.029	0.239	26.498
rs2624839	3	50202231	T	C	0.005	0.009	0.001	2.30E-07	0.026	0.728	26.790
rs2693285	2	199702908	A	G	0.005	0.017	0.001	1.00E-07	0.026	0.522	28.323
rs322640	18	34592625	A	T	-0.009	-0.021	0.002	1.10E-07	0.044	0.632	28.271
rs34364357	3	140346921	A	T	-0.014	0.104	0.003	2.90E-07	0.071	0.142	26.291
rs35566160	2	164928199	A	G	0.006	0.033	0.001	4.50E-08	0.029	0.255	29.927
rs36072649	4	140939110	T	A	-0.007	-0.022	0.001	1.10E-11	0.026	0.409	46.094
rs4551449	9	22610366	C	T	-0.008	-0.009	0.001	6.80E-08	0.036	0.805	29.116
rs4865667	5	50748173	C	T	-0.006	-0.022	0.001	3.40E-08	0.026	0.403	30.451
rs55741516	14	42697579	T	C	-0.005	0.009	0.001	2.90E-07	0.027	0.744	26.309
rs55876337	2	137399539	C	A	0.007	-0.008	0.001	1.10E-07	0.032	0.800	28.276
rs56322375	17	32906373	G	T	0.007	0.054	0.001	9.40E-08	0.031	0.078	28.493
rs576982	15	78870803	C	T	-0.009	-0.078	0.001	2.30E-14	0.031	0.011	58.275
rs6011779	20	61984317	C	T	-0.010	0.003	0.001	2.50E-14	0.033	0.921	58.089
rs62259104	3	67116763	T	C	0.010	0.086	0.002	1.40E-07	0.046	0.061	27.694
rs62477310	7	114951541	T	C	-0.006	-0.038	0.001	2.00E-08	0.026	0.136	31.496
rs7002049	8	93114414	T	C	0.008	-0.016	0.001	1.40E-09	0.031	0.609	36.620
rs72733290	1	193723842	G	A	-0.008	-0.001	0.002	2.10E-07	0.040	0.975	26.932
rs72783734	5	86811301	C	A	-0.009	0.038	0.002	3.10E-07	0.044	0.386	26.163
rs74956912	2	162878335	G	A	-0.010	0.046	0.002	5.00E-07	0.052	0.375	25.256
rs75596189	9	136468701	C	T	0.012	0.060	0.002	2.10E-13	0.041	0.145	53.864
rs78509373	17	43266542	T	C	0.007	0.034	0.001	3.50E-07	0.034	0.320	25.936
rs7899608	10	104727304	C	T	0.009	0.016	0.001	2.30E-09	0.037	0.655	35.667
rs792458	10	22133793	T	G	-0.006	-0.064	0.001	1.20E-07	0.028	0.022	27.957
rs794356	7	75196531	G	A	-0.006	-0.004	0.001	1.90E-08	0.027	0.890	31.546

Chr., Chromosome; exp, exposure; out, outcome, SE, Standard Error; SNP, Single Nucleotide Polymorphism.

Table S4. Characteristics of the genetic variants associated with maternal smoking around birth and COVID-19.

SNP	Chr.	Position	Effect allele	Other allele	ß_exp	ß_out	SE_exp	Pvalue_exp	SE_out	Pvalue_out	Fvalue
rs10135248	14	104122310	T	C	-0.005	-0.010	0.001	2.20E-07	0.010	0.287	26.848
rs10226228	7	32315613	A	G	0.007	0.002	0.001	4.00E-12	0.010	0.822	48.137
rs113331826	11	29861212	T	C	-0.018	-0.021	0.003	1.10E-07	0.032	0.522	28.271
rs12089815	1	91189933	G	A	-0.005	-0.010	0.001	4.10E-07	0.010	0.294	25.668
rs12137221	1	50182298	C	T	0.006	0.007	0.001	2.70E-07	0.012	0.555	26.473
rs12203592	6	396321	C	T	0.006	0.039	0.001	4.80E-07	0.014	0.005	25.335
rs12405972	1	44097438	G	T	-0.008	-0.012	0.001	6.40E-14	0.010	0.225	56.247
rs12923476	16	24798079	G	A	-0.007	-0.008	0.001	4.80E-09	0.011	0.463	34.260
rs13123903	4	35513601	C	T	0.005	0.002	0.001	3.80E-07	0.009	0.849	25.814
rs13130893	4	46983522	A	G	0.006	0.000	0.001	1.00E-07	0.010	0.995	28.327
rs1323341	9	14453010	A	G	-0.007	0.007	0.001	3.90E-08	0.011	0.516	30.203
rs144200291	3	51921338	G	A	-0.022	-0.029	0.004	5.00E-07	0.038	0.450	25.270
rs1569216	4	172473450	A	G	0.005	-0.008	0.001	4.40E-07	0.011	0.442	25.520
rs17078	10	106653311	A	G	-0.007	0.010	0.001	2.00E-07	0.012	0.389	27.028
rs17133347	7	3472721	C	T	-0.009	0.019	0.002	9.00E-08	0.015	0.215	28.577
rs17343902	5	103677751	A	G	-0.007	0.004	0.001	3.30E-07	0.013	0.762	26.053
rs174375	6	111908266	C	G	-0.006	-0.013	0.001	1.20E-07	0.010	0.217	27.986
rs2183947	6	26159356	G	A	-0.008	-0.020	0.001	1.50E-10	0.012	0.092	40.977
rs2428019	11	113678423	C	A	0.007	0.003	0.001	5.10E-09	0.012	0.785	34.158
rs254945	16	8956703	G	A	-0.006	-0.007	0.001	2.60E-07	0.012	0.558	26.498
rs2624839	3	50202231	T	C	0.005	0.015	0.001	2.30E-07	0.010	0.121	26.790
rs2693285	2	199702908	A	G	0.005	-0.005	0.001	1.00E-07	0.010	0.575	28.323
rs322640	18	34592625	A	T	-0.009	-0.034	0.002	1.10E-07	0.016	0.038	28.271
rs34364357	3	140346921	A	T	-0.014	0.033	0.003	2.90E-07	0.028	0.240	26.291
rs35566160	2	164928199	A	G	0.006	-0.024	0.001	4.50E-08	0.011	0.034	29.927
rs36072649	4	140939110	T	A	-0.007	-0.010	0.001	1.10E-11	0.010	0.300	46.094
rs4551449	9	22610366	C	T	-0.008	0.003	0.001	6.80E-08	0.013	0.841	29.116
rs4865667	5	50748173	C	T	-0.006	-0.011	0.001	3.40E-08	0.010	0.280	30.451
rs55741516	14	42697579	T	C	-0.005	-0.005	0.001	2.90E-07	0.010	0.625	26.309
rs55876337	2	137399539	C	A	0.007	0.004	0.001	1.10E-07	0.013	0.748	28.276
rs56322375	17	32906373	G	T	0.007	0.007	0.001	9.40E-08	0.012	0.570	28.493
rs576982	15	78870803	C	T	-0.009	-0.004	0.001	2.30E-14	0.011	0.693	58.275
rs6011779	20	61984317	C	T	-0.010	0.014	0.001	2.50E-14	0.012	0.265	58.089
rs62259104	3	67116763	T	C	0.010	0.014	0.002	1.40E-07	0.018	0.423	27.694
rs62477310	7	114951541	T	C	-0.006	0.000	0.001	2.00E-08	0.010	1.000	31.496
rs7002049	8	93114414	T	C	0.008	0.033	0.001	1.40E-09	0.013	0.009	36.620
rs72733290	1	193723842	G	A	-0.008	-0.010	0.002	2.10E-07	0.015	0.487	26.932
rs72783734	5	86811301	C	A	-0.009	-0.007	0.002	3.10E-07	0.016	0.671	26.163
rs74956912	2	162878335	G	A	-0.010	0.002	0.002	5.00E-07	0.020	0.938	25.256
rs75596189	9	136468701	C	T	0.012	0.002	0.002	2.10E-13	0.016	0.879	53.864
rs78509373	17	43266542	T	C	0.007	0.006	0.001	3.50E-07	0.013	0.638	25.936
rs7899608	10	104727304	C	T	0.009	0.027	0.001	2.30E-09	0.013	0.041	35.667
rs792458	10	22133793	T	G	-0.006	0.012	0.001	1.20E-07	0.011	0.278	27.957
rs794356	7	75196531	G	A	-0.006	-0.005	0.001	1.90E-08	0.011	0.628	31.546

Chr., Chromosome; exp, exposure; out, outcome, SE, Standard Error; SNP, Single Nucleotide Polymorphism; COVID-19, Coronavirus Disease 2019.

Table S5. Characteristics of the genetic variants associated with maternal smoking around birth and acute bronchitis.

SNP	Chr.	Position	Effect allele	Other allele	ß_exp	ß_out	SE_exp	Pvalue_exp	SE_out	Pvalue_out	Fvalue
rs10135248	14	104122310	T	C	-0.005	-0.033	0.001	2.20E-07	0.018	0.073	26.848
rs10226228	7	32315613	A	G	0.007	-0.017	0.001	4.00E-12	0.019	0.365	48.137
rs113331826	11	29861212	T	C	-0.018	-0.025	0.003	1.10E-07	0.062	0.687	28.271
rs12089815	1	91189933	G	A	-0.005	0.011	0.001	4.10E-07	0.018	0.550	25.668
rs12137221	1	50182298	C	T	0.006	-0.008	0.001	2.70E-07	0.021	0.717	26.473
rs12203592	6	396321	C	T	0.006	0.086	0.001	4.80E-07	0.051	0.090	25.335
rs12405972	1	44097438	G	T	-0.008	0.004	0.001	6.40E-14	0.018	0.824	56.247
rs12923476	16	24798079	G	A	-0.007	-0.019	0.001	4.80E-09	0.021	0.359	34.260
rs13123903	4	35513601	C	T	0.005	0.025	0.001	3.80E-07	0.017	0.141	25.814
rs13130893	4	46983522	A	G	0.006	-0.009	0.001	1.00E-07	0.019	0.630	28.327
rs1323341	9	14453010	A	G	-0.007	-0.049	0.001	3.90E-08	0.019	0.012	30.203
rs144200291	3	51921338	G	A	-0.022	0.034	0.004	5.00E-07	0.060	0.573	25.270
rs1569216	4	172473450	A	G	0.005	0.029	0.001	4.40E-07	0.018	0.113	25.520
rs17078	10	106653311	A	G	-0.007	-0.034	0.001	2.00E-07	0.020	0.083	27.028
rs17133347	7	3472721	C	T	-0.009	-0.048	0.002	9.00E-08	0.019	0.013	28.577
rs17343902	5	103677751	A	G	-0.007	-0.015	0.001	3.30E-07	0.021	0.482	26.053
rs174375	6	111908266	C	G	-0.006	-0.021	0.001	1.20E-07	0.018	0.241	27.986
rs2183947	6	26159356	G	A	-0.008	-0.050	0.001	1.50E-10	0.020	0.014	40.977
rs2428019	11	113678423	C	A	0.007	0.017	0.001	5.10E-09	0.020	0.388	34.158
rs254945	16	8956703	G	A	-0.006	0.020	0.001	2.60E-07	0.020	0.317	26.498
rs2624839	3	50202231	T	C	0.005	0.025	0.001	2.30E-07	0.018	0.158	26.790
rs2693285	2	199702908	A	G	0.005	-0.017	0.001	1.00E-07	0.017	0.311	28.323
rs322640	18	34592625	A	T	-0.009	0.003	0.002	1.10E-07	0.028	0.906	28.271
rs34364357	3	140346921	A	T	-0.014	-0.095	0.003	2.90E-07	0.076	0.208	26.291
rs35566160	2	164928199	A	G	0.006	0.002	0.001	4.50E-08	0.020	0.908	29.927
rs36072649	4	140939110	T	A	-0.007	-0.012	0.001	1.10E-11	0.018	0.489	46.094
rs4551449	9	22610366	C	T	-0.008	-0.007	0.001	6.80E-08	0.019	0.701	29.116
rs4865667	5	50748173	C	T	-0.006	0.012	0.001	3.40E-08	0.018	0.501	30.451
rs55741516	14	42697579	T	C	-0.005	-0.016	0.001	2.90E-07	0.018	0.360	26.309
rs55876337	2	137399539	C	A	0.007	-0.006	0.001	1.10E-07	0.023	0.798	28.276
rs56322375	17	32906373	G	T	0.007	-0.004	0.001	9.40E-08	0.021	0.839	28.493
rs576982	15	78870803	C	T	-0.009	-0.046	0.001	2.30E-14	0.019	0.013	58.275
rs6011779	20	61984317	C	T	-0.010	0.010	0.001	2.50E-14	0.020	0.606	58.089
rs62259104	3	67116763	T	C	0.010	0.001	0.002	1.40E-07	0.038	0.985	27.694
rs62477310	7	114951541	T	C	-0.006	-0.015	0.001	2.00E-08	0.017	0.370	31.496
rs7002049	8	93114414	T	C	0.008	0.058	0.001	1.40E-09	0.022	0.009	36.620
rs72733290	1	193723842	G	A	-0.008	-0.034	0.002	2.10E-07	0.020	0.092	26.932
rs72783734	5	86811301	C	A	-0.009	0.028	0.002	3.10E-07	0.032	0.388	26.163
rs74956912	2	162878335	G	A	-0.010	0.001	0.002	5.00E-07	0.047	0.989	25.256
rs75596189	9	136468701	C	T	0.012	0.066	0.002	2.10E-13	0.048	0.167	53.864
rs78509373	17	43266542	T	C	0.007	0.005	0.001	3.50E-07	0.019	0.785	25.936
rs7899608	10	104727304	C	T	0.009	-0.019	0.001	2.30E-09	0.024	0.411	35.667
rs792458	10	22133793	T	G	-0.006	-0.011	0.001	1.20E-07	0.018	0.560	27.957
rs794356	7	75196531	G	A	-0.006	-0.004	0.001	1.90E-08	0.017	0.824	31.546

Chr., Chromosome; exp, exposure; out, outcome, SE, Standard Error; SNP, Single Nucleotide Polymorphism.

Table S6. Characteristics of the genetic variants associated with maternal smoking around birth and pneumonia.

SNP	Chr.	Position	Effect allele	Other allele	ß_exp	ß_out	SE_exp	Pvalue_exp	SE_out	Pvalue_out	Fvalue
rs10135248	14	104122310	T	C	-0.005	-0.012	0.001	2.20E-07	0.010	0.265	26.848
rs10226228	7	32315613	A	G	0.007	-0.003	0.001	4.00E-12	0.011	0.758	48.137
rs113331826	11	29861212	T	C	-0.018	-0.005	0.003	1.10E-07	0.035	0.891	28.271
rs12089815	1	91189933	G	A	-0.005	-0.005	0.001	4.10E-07	0.010	0.613	25.668
rs12137221	1	50182298	C	T	0.006	-0.003	0.001	2.70E-07	0.012	0.826	26.473
rs12203592	6	396321	C	T	0.006	-0.027	0.001	4.80E-07	0.029	0.353	25.335
rs12405972	1	44097438	G	T	-0.008	0.004	0.001	6.40E-14	0.010	0.686	56.247
rs12923476	16	24798079	G	A	-0.007	-0.007	0.001	4.80E-09	0.012	0.535	34.260
rs13123903	4	35513601	C	T	0.005	0.026	0.001	3.80E-07	0.010	0.007	25.814
rs13130893	4	46983522	A	G	0.006	-0.009	0.001	1.00E-07	0.010	0.386	28.327
rs1323341	9	14453010	A	G	-0.007	0.004	0.001	3.90E-08	0.011	0.723	30.203
rs144200291	3	51921338	G	A	-0.022	0.002	0.004	5.00E-07	0.034	0.946	25.270
rs1569216	4	172473450	A	G	0.005	-0.001	0.001	4.40E-07	0.010	0.902	25.520
rs17078	10	106653311	A	G	-0.007	-0.007	0.001	2.00E-07	0.011	0.548	27.028
rs17133347	7	3472721	C	T	-0.009	0.004	0.002	9.00E-08	0.011	0.735	28.577
rs17343902	5	103677751	A	G	-0.007	-0.013	0.001	3.30E-07	0.012	0.283	26.053
rs174375	6	111908266	C	G	-0.006	-0.004	0.001	1.20E-07	0.010	0.686	27.986
rs2183947	6	26159356	G	A	-0.008	0.000	0.001	1.50E-10	0.011	0.991	40.977
rs2428019	11	113678423	C	A	0.007	0.003	0.001	5.10E-09	0.011	0.765	34.158
rs254945	16	8956703	G	A	-0.006	0.001	0.001	2.60E-07	0.011	0.914	26.498
rs2624839	3	50202231	T	C	0.005	-0.005	0.001	2.30E-07	0.010	0.633	26.790
rs2693285	2	199702908	A	G	0.005	-0.001	0.001	1.00E-07	0.010	0.939	28.323
rs322640	18	34592625	A	T	-0.009	-0.003	0.002	1.10E-07	0.016	0.873	28.271
rs34364357	3	140346921	A	T	-0.014	0.013	0.003	2.90E-07	0.043	0.769	26.291
rs35566160	2	164928199	A	G	0.006	0.010	0.001	4.50E-08	0.011	0.359	29.927
rs36072649	4	140939110	T	A	-0.007	-0.019	0.001	1.10E-11	0.010	0.056	46.094
rs4551449	9	22610366	C	T	-0.008	0.009	0.001	6.80E-08	0.011	0.408	29.116
rs4865667	5	50748173	C	T	-0.006	0.016	0.001	3.40E-08	0.010	0.107	30.451
rs55741516	14	42697579	T	C	-0.005	-0.013	0.001	2.90E-07	0.010	0.195	26.309
rs55876337	2	137399539	C	A	0.007	0.007	0.001	1.10E-07	0.013	0.593	28.276
rs56322375	17	32906373	G	T	0.007	0.021	0.001	9.40E-08	0.012	0.083	28.493
rs576982	15	78870803	C	T	-0.009	-0.038	0.001	2.30E-14	0.011	0.000	58.275
rs6011779	20	61984317	C	T	-0.010	-0.024	0.001	2.50E-14	0.011	0.032	58.089
rs62259104	3	67116763	T	C	0.010	-0.003	0.002	1.40E-07	0.021	0.883	27.694
rs62477310	7	114951541	T	C	-0.006	0.010	0.001	2.00E-08	0.010	0.289	31.496
rs7002049	8	93114414	T	C	0.008	-0.008	0.001	1.40E-09	0.012	0.494	36.620
rs72733290	1	193723842	G	A	-0.008	-0.019	0.002	2.10E-07	0.011	0.104	26.932
rs72783734	5	86811301	C	A	-0.009	0.004	0.002	3.10E-07	0.018	0.816	26.163
rs74956912	2	162878335	G	A	-0.010	0.015	0.002	5.00E-07	0.027	0.565	25.256
rs75596189	9	136468701	C	T	0.012	0.068	0.002	2.10E-13	0.027	0.012	53.864
rs78509373	17	43266542	T	C	0.007	0.022	0.001	3.50E-07	0.011	0.040	25.936
rs7899608	10	104727304	C	T	0.009	0.026	0.001	2.30E-09	0.013	0.055	35.667
rs792458	10	22133793	T	G	-0.006	-0.008	0.001	1.20E-07	0.010	0.441	27.957
rs794356	7	75196531	G	A	-0.006	-0.004	0.001	1.90E-08	0.010	0.713	31.546

Chr., Chromosome; exp, exposure; out, outcome, SE, Standard Error; SNP, Single Nucleotide Polymorphism.

Table S7. Characteristics of the genetic variants associated with maternal smoking around birth and bacterial pneumonia.

SNP	Chr.	Position	Effect allele	Other allele	ß_exp	ß_out	SE_exp	Pvalue_exp	SE_out	Pvalue_out	Fvalue
rs10135248	14	104122310	T	C	-0.005	-0.022	0.001	2.20E-07	0.018	0.219	26.848
rs10226228	7	32315613	A	G	0.007	-0.020	0.001	4.00E-12	0.019	0.284	48.137
rs113331826	11	29861212	T	C	-0.018	0.011	0.003	1.10E-07	0.061	0.859	28.271
rs12089815	1	91189933	G	A	-0.005	-0.022	0.001	4.10E-07	0.017	0.201	25.668
rs12137221	1	50182298	C	T	0.006	-0.013	0.001	2.70E-07	0.020	0.521	26.473
rs12203592	6	396321	C	T	0.006	-0.013	0.001	4.80E-07	0.051	0.802	25.335
rs12405972	1	44097438	G	T	-0.008	0.003	0.001	6.40E-14	0.018	0.861	56.247
rs12923476	16	24798079	G	A	-0.007	-0.007	0.001	4.80E-09	0.020	0.721	34.260
rs13123903	4	35513601	C	T	0.005	0.029	0.001	3.80E-07	0.017	0.079	25.814
rs13130893	4	46983522	A	G	0.006	0.008	0.001	1.00E-07	0.018	0.677	28.327
rs1323341	9	14453010	A	G	-0.007	0.005	0.001	3.90E-08	0.019	0.804	30.203
rs144200291	3	51921338	G	A	-0.022	0.000	0.004	5.00E-07	0.059	0.997	25.270
rs1569216	4	172473450	A	G	0.005	-0.021	0.001	4.40E-07	0.018	0.245	25.520
rs17078	10	106653311	A	G	-0.007	-0.005	0.001	2.00E-07	0.019	0.800	27.028
rs17133347	7	3472721	C	T	-0.009	-0.007	0.002	9.00E-08	0.019	0.712	28.577
rs17343902	5	103677751	A	G	-0.007	-0.031	0.001	3.30E-07	0.020	0.120	26.053
rs174375	6	111908266	C	G	-0.006	0.002	0.001	1.20E-07	0.017	0.900	27.986
rs2183947	6	26159356	G	A	-0.008	-0.001	0.001	1.50E-10	0.020	0.954	40.977
rs2428019	11	113678423	C	A	0.007	-0.001	0.001	5.10E-09	0.020	0.957	34.158
rs254945	16	8956703	G	A	-0.006	-0.006	0.001	2.60E-07	0.019	0.742	26.498
rs2624839	3	50202231	T	C	0.005	-0.007	0.001	2.30E-07	0.017	0.685	26.790
rs2693285	2	199702908	A	G	0.005	0.000	0.001	1.00E-07	0.017	0.995	28.323
rs322640	18	34592625	A	T	-0.009	-0.050	0.002	1.10E-07	0.028	0.074	28.271
rs34364357	3	140346921	A	T	-0.014	-0.015	0.003	2.90E-07	0.077	0.842	26.291
rs35566160	2	164928199	A	G	0.006	0.023	0.001	4.50E-08	0.019	0.232	29.927
rs36072649	4	140939110	T	A	-0.007	-0.038	0.001	1.10E-11	0.017	0.029	46.094
rs4551449	9	22610366	C	T	-0.008	0.031	0.001	6.80E-08	0.019	0.104	29.116
rs4865667	5	50748173	C	T	-0.006	-0.007	0.001	3.40E-08	0.017	0.685	30.451
rs55741516	14	42697579	T	C	-0.005	-0.014	0.001	2.90E-07	0.018	0.419	26.309
rs55876337	2	137399539	C	A	0.007	0.022	0.001	1.10E-07	0.023	0.328	28.276
rs56322375	17	32906373	G	T	0.007	0.035	0.001	9.40E-08	0.021	0.098	28.493
rs576982	15	78870803	C	T	-0.009	-0.045	0.001	2.30E-14	0.018	0.015	58.275
rs6011779	20	61984317	C	T	-0.010	-0.023	0.001	2.50E-14	0.020	0.247	58.089
rs62259104	3	67116763	T	C	0.010	0.038	0.002	1.40E-07	0.037	0.300	27.694
rs62477310	7	114951541	T	C	-0.006	-0.003	0.001	2.00E-08	0.017	0.864	31.496
rs7002049	8	93114414	T	C	0.008	-0.032	0.001	1.40E-09	0.022	0.134	36.620
rs72733290	1	193723842	G	A	-0.008	-0.038	0.002	2.10E-07	0.020	0.054	26.932
rs72783734	5	86811301	C	A	-0.009	0.018	0.002	3.10E-07	0.032	0.565	26.163
rs74956912	2	162878335	G	A	-0.010	-0.023	0.002	5.00E-07	0.048	0.623	25.256
rs75596189	9	136468701	C	T	0.012	0.017	0.002	2.10E-13	0.048	0.721	53.864
rs78509373	17	43266542	T	C	0.007	0.036	0.001	3.50E-07	0.019	0.057	25.936
rs7899608	10	104727304	C	T	0.009	0.035	0.001	2.30E-09	0.023	0.136	35.667
rs792458	10	22133793	T	G	-0.006	0.005	0.001	1.20E-07	0.018	0.772	27.957
rs794356	7	75196531	G	A	-0.006	0.017	0.001	1.90E-08	0.017	0.328	31.546

Chr., Chromosome; exp, exposure; out, outcome, SE, Standard Error; SNP, Single Nucleotide Polymorphism.

Table S8. Characteristics of the genetic variants associated with maternal smoking around birth and viral pneumonia.

SNP	Chr.	Position	Effect allele	Other allele	ß_exp	ß_out	SE_exp	Pvalue_exp	SE_out	Pvalue_out	Fvalue
rs10135248	14	104122310	T	C	-0.005	0.017	0.001	2.20E-07	0.059	0.770	26.848
rs10226228	7	32315613	A	G	0.007	0.037	0.001	4.00E-12	0.060	0.540	48.137
rs113331826	11	29861212	T	C	-0.018	-0.143	0.003	1.10E-07	0.198	0.470	28.271
rs12089815	1	91189933	G	A	-0.005	0.006	0.001	4.10E-07	0.056	0.908	25.668
rs12137221	1	50182298	C	T	0.006	0.090	0.001	2.70E-07	0.066	0.169	26.473
rs12203592	6	396321	C	T	0.006	-0.076	0.001	4.80E-07	0.161	0.638	25.335
rs12405972	1	44097438	G	T	-0.008	-0.001	0.001	6.40E-14	0.056	0.992	56.247
rs12923476	16	24798079	G	A	-0.007	-0.016	0.001	4.80E-09	0.066	0.812	34.260
rs13123903	4	35513601	C	T	0.005	0.145	0.001	3.80E-07	0.054	0.007	25.814
rs13130893	4	46983522	A	G	0.006	-0.007	0.001	1.00E-07	0.059	0.900	28.327
rs1323341	9	14453010	A	G	-0.007	0.064	0.001	3.90E-08	0.062	0.297	30.203
rs144200291	3	51921338	G	A	-0.022	0.192	0.004	5.00E-07	0.190	0.312	25.270
rs1569216	4	172473450	A	G	0.005	0.067	0.001	4.40E-07	0.058	0.248	25.520
rs17078	10	106653311	A	G	-0.007	-0.038	0.001	2.00E-07	0.063	0.544	27.028
rs17133347	7	3472721	C	T	-0.009	-0.062	0.002	9.00E-08	0.061	0.309	28.577
rs17343902	5	103677751	A	G	-0.007	0.037	0.001	3.30E-07	0.066	0.570	26.053
rs174375	6	111908266	C	G	-0.006	0.042	0.001	1.20E-07	0.056	0.447	27.986
rs2183947	6	26159356	G	A	-0.008	0.003	0.001	1.50E-10	0.064	0.963	40.977
rs2428019	11	113678423	C	A	0.007	-0.093	0.001	5.10E-09	0.064	0.146	34.158
rs254945	16	8956703	G	A	-0.006	0.076	0.001	2.60E-07	0.062	0.221	26.498
rs2624839	3	50202231	T	C	0.005	-0.019	0.001	2.30E-07	0.056	0.733	26.790
rs2693285	2	199702908	A	G	0.005	0.008	0.001	1.00E-07	0.054	0.887	28.323
rs322640	18	34592625	A	T	-0.009	0.073	0.002	1.10E-07	0.089	0.410	28.271
rs34364357	3	140346921	A	T	-0.014	0.185	0.003	2.90E-07	0.242	0.445	26.291
rs35566160	2	164928199	A	G	0.006	0.067	0.001	4.50E-08	0.062	0.280	29.927
rs36072649	4	140939110	T	A	-0.007	0.057	0.001	1.10E-11	0.056	0.312	46.094
rs4551449	9	22610366	C	T	-0.008	0.007	0.001	6.80E-08	0.061	0.903	29.116
rs4865667	5	50748173	C	T	-0.006	0.031	0.001	3.40E-08	0.056	0.578	30.451
rs55741516	14	42697579	T	C	-0.005	-0.036	0.001	2.90E-07	0.057	0.530	26.309
rs55876337	2	137399539	C	A	0.007	0.050	0.001	1.10E-07	0.074	0.498	28.276
rs56322375	17	32906373	G	T	0.007	-0.042	0.001	9.40E-08	0.068	0.538	28.493
rs576982	15	78870803	C	T	-0.009	-0.084	0.001	2.30E-14	0.060	0.159	58.275
rs6011779	20	61984317	C	T	-0.010	0.033	0.001	2.50E-14	0.063	0.604	58.089
rs62259104	3	67116763	T	C	0.010	0.073	0.002	1.40E-07	0.120	0.542	27.694
rs62477310	7	114951541	T	C	-0.006	0.081	0.001	2.00E-08	0.054	0.137	31.496
rs7002049	8	93114414	T	C	0.008	0.068	0.001	1.40E-09	0.069	0.328	36.620
rs72733290	1	193723842	G	A	-0.008	0.010	0.002	2.10E-07	0.065	0.882	26.932
rs72783734	5	86811301	C	A	-0.009	0.007	0.002	3.10E-07	0.103	0.946	26.163
rs74956912	2	162878335	G	A	-0.010	-0.002	0.002	5.00E-07	0.151	0.991	25.256
rs75596189	9	136468701	C	T	0.012	0.084	0.002	2.10E-13	0.153	0.582	53.864
rs78509373	17	43266542	T	C	0.007	-0.024	0.001	3.50E-07	0.062	0.695	25.936
rs7899608	10	104727304	C	T	0.009	0.021	0.001	2.30E-09	0.075	0.784	35.667
rs792458	10	22133793	T	G	-0.006	-0.060	0.001	1.20E-07	0.058	0.301	27.957
rs794356	7	75196531	G	A	-0.006	-0.098	0.001	1.90E-08	0.055	0.074	31.546

Chr., Chromosome; exp, exposure; out, outcome, SE, Standard Error; SNP, Single Nucleotide Polymorphism.

Table S9. Characteristics of the genetic variants associated with maternal smoking around birth and influenza pneumonia.

SNP	Chr.	Position	Effect allele	Other allele	ß_exp	ß_out	SE_exp	Pvalue_exp	SE_out	Pvalue_out	Fvalue
rs10135248	14	104122310	T	C	-0.005	-0.009	0.001	2.20E-07	0.010	0.357	26.848
rs10226228	7	32315613	A	G	0.007	0.002	0.001	4.00E-12	0.010	0.853	48.137
rs113331826	11	29861212	T	C	-0.018	-0.018	0.003	1.10E-07	0.033	0.601	28.271
rs12089815	1	91189933	G	A	-0.005	-0.003	0.001	4.10E-07	0.010	0.745	25.668
rs12137221	1	50182298	C	T	0.006	-0.008	0.001	2.70E-07	0.011	0.488	26.473
rs12203592	6	396321	C	T	0.006	-0.005	0.001	4.80E-07	0.028	0.849	25.335
rs12405972	1	44097438	G	T	-0.008	0.009	0.001	6.40E-14	0.010	0.332	56.247
rs12923476	16	24798079	G	A	-0.007	-0.003	0.001	4.80E-09	0.011	0.779	34.260
rs13123903	4	35513601	C	T	0.005	0.026	0.001	3.80E-07	0.009	0.005	25.814
rs13130893	4	46983522	A	G	0.006	-0.010	0.001	1.00E-07	0.010	0.300	28.327
rs1323341	9	14453010	A	G	-0.007	0.006	0.001	3.90E-08	0.011	0.582	30.203
rs144200291	3	51921338	G	A	-0.022	0.009	0.004	5.00E-07	0.032	0.787	25.270
rs1569216	4	172473450	A	G	0.005	-0.001	0.001	4.40E-07	0.010	0.890	25.520
rs17078	10	106653311	A	G	-0.007	-0.011	0.001	2.00E-07	0.011	0.309	27.028
rs17133347	7	3472721	C	T	-0.009	0.006	0.002	9.00E-08	0.010	0.568	28.577
rs17343902	5	103677751	A	G	-0.007	-0.017	0.001	3.30E-07	0.011	0.121	26.053
rs174375	6	111908266	C	G	-0.006	-0.003	0.001	1.20E-07	0.010	0.793	27.986
rs2183947	6	26159356	G	A	-0.008	0.002	0.001	1.50E-10	0.011	0.847	40.977
rs2428019	11	113678423	C	A	0.007	0.002	0.001	5.10E-09	0.011	0.885	34.158
rs254945	16	8956703	G	A	-0.006	-0.004	0.001	2.60E-07	0.011	0.699	26.498
rs2624839	3	50202231	T	C	0.005	-0.001	0.001	2.30E-07	0.010	0.888	26.790
rs2693285	2	199702908	A	G	0.005	-0.003	0.001	1.00E-07	0.009	0.783	28.323
rs322640	18	34592625	A	T	-0.009	0.005	0.002	1.10E-07	0.015	0.752	28.271
rs34364357	3	140346921	A	T	-0.014	0.030	0.003	2.90E-07	0.041	0.471	26.291
rs35566160	2	164928199	A	G	0.006	0.014	0.001	4.50E-08	0.011	0.201	29.927
rs36072649	4	140939110	T	A	-0.007	-0.017	0.001	1.10E-11	0.010	0.086	46.094
rs4551449	9	22610366	C	T	-0.008	0.013	0.001	6.80E-08	0.010	0.230	29.116
rs4865667	5	50748173	C	T	-0.006	0.015	0.001	3.40E-08	0.010	0.111	30.451
rs55741516	14	42697579	T	C	-0.005	-0.013	0.001	2.90E-07	0.010	0.198	26.309
rs55876337	2	137399539	C	A	0.007	0.003	0.001	1.10E-07	0.013	0.792	28.276
rs56322375	17	32906373	G	T	0.007	0.024	0.001	9.40E-08	0.012	0.036	28.493
rs576982	15	78870803	C	T	-0.009	-0.036	0.001	2.30E-14	0.010	0.000	58.275
rs6011779	20	61984317	C	T	-0.010	-0.018	0.001	2.50E-14	0.011	0.104	58.089
rs62259104	3	67116763	T	C	0.010	-0.011	0.002	1.40E-07	0.020	0.593	27.694
rs62477310	7	114951541	T	C	-0.006	0.003	0.001	2.00E-08	0.009	0.738	31.496
rs7002049	8	93114414	T	C	0.008	-0.005	0.001	1.40E-09	0.012	0.705	36.620
rs72733290	1	193723842	G	A	-0.008	-0.014	0.002	2.10E-07	0.011	0.204	26.932
rs72783734	5	86811301	C	A	-0.009	0.001	0.002	3.10E-07	0.018	0.977	26.163
rs74956912	2	162878335	G	A	-0.010	0.024	0.002	5.00E-07	0.026	0.357	25.256
rs75596189	9	136468701	C	T	0.012	0.062	0.002	2.10E-13	0.026	0.017	53.864
rs78509373	17	43266542	T	C	0.007	0.022	0.001	3.50E-07	0.011	0.038	25.936
rs7899608	10	104727304	C	T	0.009	0.026	0.001	2.30E-09	0.013	0.041	35.667
rs792458	10	22133793	T	G	-0.006	-0.004	0.001	1.20E-07	0.010	0.686	27.957
rs794356	7	75196531	G	A	-0.006	-0.004	0.001	1.90E-08	0.009	0.697	31.546

Chr., Chromosome; exp, exposure; out, outcome, SE, Standard Error; SNP, Single Nucleotide Polymorphism.
